# Self-organization in Balanced State Networks by STDP and Homeostatic Plasticity

**DOI:** 10.1371/journal.pcbi.1004420

**Published:** 2015-09-03

**Authors:** Felix Effenberger, Jürgen Jost, Anna Levina

**Affiliations:** 1 Max-Planck-Institute for Mathematics in the Sciences, Leipzig, Germany; 2 Bernstein Center for Computational Neuroscience Göttingen, Göttingen, Germany; Research Center Jülich, GERMANY

## Abstract

Structural inhomogeneities in synaptic efficacies have a strong impact on population response dynamics of cortical networks and are believed to play an important role in their functioning. However, little is known about how such inhomogeneities could evolve by means of synaptic plasticity. Here we present an adaptive model of a balanced neuronal network that combines two different types of plasticity, STDP and synaptic scaling. The plasticity rules yield both long-tailed distributions of synaptic weights and firing rates. Simultaneously, a highly connected subnetwork of *driver neurons* with strong synapses emerges. Coincident spiking activity of several driver cells can evoke population bursts and driver cells have similar dynamical properties as leader neurons found experimentally. Our model allows us to observe the delicate interplay between structural and dynamical properties of the emergent inhomogeneities. It is simple, robust to parameter changes and able to explain a multitude of different experimental findings in one basic network.

## Author Summary

It is widely believed that the structure of neuronal circuits plays a major role in brain functioning. Although the full synaptic connectivity for larger populations is not yet assessable even by current experimental techniques, available data show that neither synaptic strengths nor the number of synapses per neuron are homogeneously distributed. Several studies have found long-tailed distributions of synaptic weights with many weak and a few exceptionally strong synaptic connections, as well as strongly connected cells and subnetworks that may play a decisive role for data processing in neural circuits. Little is known about how inhomogeneities could arise in the developing brain and we hypothesize that there is a self-organizing principle behind their appearance. In this study we show how structural inhomogeneities can emerge by simple synaptic plasticity mechanisms from an initially homogeneous network. We perform numerical simulations and show analytically how a small imbalance in the initial structure is amplified by the synaptic plasticities and their interplay. Our network can simultaneously explain several experimental observations that were previously not linked.

## Introduction

Distributions of synaptic weights are known to have a large influence on the dynamics and information-processing properties of neural circuits [1–5]. Recent electrophysiological studies have shown that the distributions of the amplitudes of excitatory postsynaptic potentials (EPSPs) in cortical [6, 7] as well as hippocampal [5] networks are typically long-tailed and span several orders of magnitude. This characterizes a topological configuration in which the majority of synaptic connections are weak and a small number are much stronger [6–9]. At the same time, distributions of firing rates during spontaneous activity have been found to be long-tailed [10] and there is increasing evidence that long-tailed distributions play a fundamental role in brain functioning [11–13].

Studies of microcircuit connectivity have also demonstrated a number of significant inhomogeneities, as well as correlations amongst cell activity. Fine-scale functional subnetworks have been found in cortical networks [1, 3, 14], and it has been shown that cells with strong outgoing synapses cluster together [3, 7]. Both the clustering of the highly active cells [3] and the presence of strong synapses [1] are likely to play an important role for network dynamics and information processing in the neocortex.

Apart from structural inhomogeneities in networks, the impact of individual neurons on the dynamics of neural networks may also differ substantially. A number of recent *in vitro* studies of 1D and 2D dissociated developing cortical and hippocampal cultures have shown that such networks typically express spontaneous neural activity characterized by network bursts, and ongoing repetitions of distinctive firing patterns within those bursts [15–19]. Furthermore, several studies have shown that the activity of certain neurons reliably precedes population spikes [16–19]. These early-to-fire neurons have been termed *leader neurons* [18] and have been found to form functionally connected networks, the activity of which collectively precedes most of the observed population bursts [19]. In the 1D case, population bursts have been found to be triggered by “burst initiation zones” [17] and in the 2D case recent studies [18, 20] have shown that leader neurons not only precede but also are able to initiate population bursts. Nevertheless, the underlying network structure and specific topological properties of leader neurons and subnetworks of such cells remain to be discovered. Experimental studies in constrained [21] or chemically modulated [22] cultures give reason to believe that a complicated process of self-organization underlies their emergence. However, from the modeling point of view, little is understood about how strong inhomogeneities, such as the aforementioned, could evolve in a self-organized manner by means of activity-dependent synaptic plasticity.

Synaptic plasticity is widely believed to be the basis for learning and memory and shapes the distribution of synaptic weights, which has been found to be typically long-tailed in a number of recent experimental studies [6, 7]. The influence of long-tailed weight distributions on network dynamics has been studied in a number of recent works [2, 4, 5, 12, 23], and it has been shown that such distributions can increase spike-based information transfer and facilitate information processing in neural networks [5, 24].

Yet, also in the latter case little is known about how experimentally observed activity-modulated synaptic plasticity, such as spike-timing-dependent plasticity [25–27] (STDP) and homeostatic plasticity [28, 29] could lead to a symmetry-breaking in the distributions of synaptic weights of an initially homogeneous network, or to the preservation of inhomogeneities prescribed by certain initial conditions [30, 31]. Even less is known about how networks could self-organize to simultaneously express the aforementioned properties such as having both long-tailed distributions of weights and firing rates at the same time [2], cells with different dynamical effects on network activity, and subnetworks with distinct dynamical and structural properties similar to the ones observed experimentally. Although recent theoretical models have proposed mechanisms that lead to the emergence of long-tailed synaptic weight distributions [32, 33], and in case of the SORN model [33] to other interesting aspects of self-organization, these either employ a specially tailored plasticity rule for this purpose [32], or do not express long-tailed firing-rate distributions [33].

It is well known that networks of spiking neurons can exhibit strongly irregular dynamics if excitatory and inhibitory inputs to each neuron balance, such that the network is driven by fluctuations in its input, resulting in each neuron producing spikes at irregular times [24, 34, 35]. Such networks are called *balanced state networks*. They combine computational simplicity and dynamics that closely resembles activity recorded electrophysiologically *in vivo* from cortical cells of behaving animals [36, 37]. This makes balanced state networks a very attractive and widely used theoretical model for cortical tissue [34, 35, 38, 39].

Our goal for this study was to investigate processes of self-organization in such networks brought about by means of synaptic plasticity. We therefore consider a random network of spiking neurons in the balanced state, operating in the asynchronous irregular (AI) regime [34, 35] that is believed to be a good fit to the activity of cortical networks *in vivo* [36, 37]. We endow it with two activity-dependent synaptic plasticity rules, namely spike-timing-dependent plasticity [26, 40, 41] (STDP) and synaptic scaling [29].

In its prototypical form, STDP causes long-term potentiation (LTP) of a synapse if presynaptic spikes repeatedly occur some milliseconds before postsynaptic ones, whereas a reversed temporal order causes long-term depression (LTD). Since its initial discovery at glutamatergic synapses [25, 26, 41], many forms of STDP have been observed experimentally [42], also such forms acting at GABAergic synaptic connections [43–47] and many models have been proposed [27, 48–52] to describe the mechanisms and dynamics of STDP. In particular, STDP acting at inhibitory-excitatory connections has been shown to influence spiking dynamics of hippocampal pyramidal neurons [45]. Several recent modeling studies have shown that inhibitory STDP has a stabilizing effect on network dynamics [53–56], and others also have started addressing questions of functional aspects of inhibitory plasticity [57, 58].

Another well-studied form of synaptic plasticity is synaptic scaling [29, 59], a form of homeostatic synaptic plasticity [28] that describes the up- and down-regulation of a neuron’s synaptic input in order to keep a preferred mean target firing rate.

It is well known that STDP alone can lead to instabilities in network dynamics due to effects of positive resonance which result in runaway excitation, and that endowing a random network solely with a multiplicative or power-law STDP rule acting at excitatory-excitatory synaptic connections while keeping the other synaptic efficacies fixed does not lead to stable effects of self-organization [30, 31]. Combinations of STDP and synaptic scaling, however, are known to be able to keep network dynamics in a stable and biologically plausible regime [60], and to support non-trivial computations underlying many optimization tasks [61]. Furthermore, it has been shown that combining Hebbian and homeostatic plasticity rules both has a stabilizing effect on network dynamics [62], and it has been found to express structure-building properties in simple model networks [63] of rate-based neurons.

Here, we investigate how the inclusion of synaptic scaling and inhibitory STDP could bring about self-organization in spiking networks. Our model network develops both long-tailed distributions of synaptic weights and firing rates, similar to those observed experimentally [6, 10]. Moreover, a delicate interplay between dynamics and synaptic plasticity leads to the emergence of a special group of neurons that we call *driver neurons*. They form subnetworks that can take strong influence on network dynamics and share properties of certain neurons and subnetworks found experimentally [1, 3, 18, 19]. The phenomena that we observe are generic and hold under alternations of the plasticity rules and their parameters.

## Results

We started with a classical balanced state model [34, 35] as a randomly connected, fully recurrent network of leaky integrate and fire neurons (see Methods). We first considered a static network in which all synaptic efficacies are equal and constant and we reproduced the well-studied asynchronous irregular (AI) state of activity in which neuronal spiking is fluctuation-driven. We then endowed the network with different kinds of synaptic plasticity. Specifically, we added additive Hebbian STDP rules acting at excitatory-excitatory [27] (E-E) and inhibitory-excitatory [44] (I-E) synaptic connections and a synaptic scaling rule [28, 29] that acts at the postsynaptic site of E-E connections (see Fig A in S1 Text for a schema of the network setup). The synaptic scaling is implemented as a normalization of the sum of all incoming excitatory synaptic connections, similar to experimentally observed rules [59] (see Methods).

In all which follows, synaptic weights are normalized so that their initial values are 1, and then multiplied by a scaling factor to obtain a physiological quantity (see Methods). Dynamic synapses are allowed to change in strength to values between a minimal weight of 0 and a maximal weight of $w_{\text{e}}^{\text{max}}=20$ and $w_{\text{i}}^{\text{max}}=5$ for E-E and I-E connections, respectively.

Raster plots of network activity without plasticity and after a transient phase lasting around 5 hours of network activity after switching on the plasticity rules show qualitatively similar behavior (see Fig B in S1 Text). During the transient phase the network rests in the AI state of activity and expresses long-tailed distributions of firing rates. We observe that synaptic plasticity increases the mean population firing rates, but only very slightly.

Apart from networks in which all initial weights are equal, we also considered networks with initially Gaussian and uniform weight distributions and obtained the same qualitative results after a transient phase. We also found that the results are not sensitive to variations in the learning rates of the STDP rules (see Section STDP learning rates). Furthermore, results remained qualitatively comparable even when replacing the learning rules (see Section Generality of the model): We studied a variant of the model in which we replaced the synaptic normalization with a homeostatic mechanism acting multiplicatively on the synaptic weights on slower time scales [28] (see Section Different forms of homeostatic plasticity and Section 12.3 in S1 Text), as well as variants in which the additive STDP rule at excitatory synapses was replaced with a partly or fully multiplicative one (see Section Different STDP rules and Section 12.1, Section 12.2 in S1 Text). For all variants we studied, most aspects of the findings were preserved (see Section Generality of the model).

### Long tailed distributions of weights and rates

**Fig 1:**
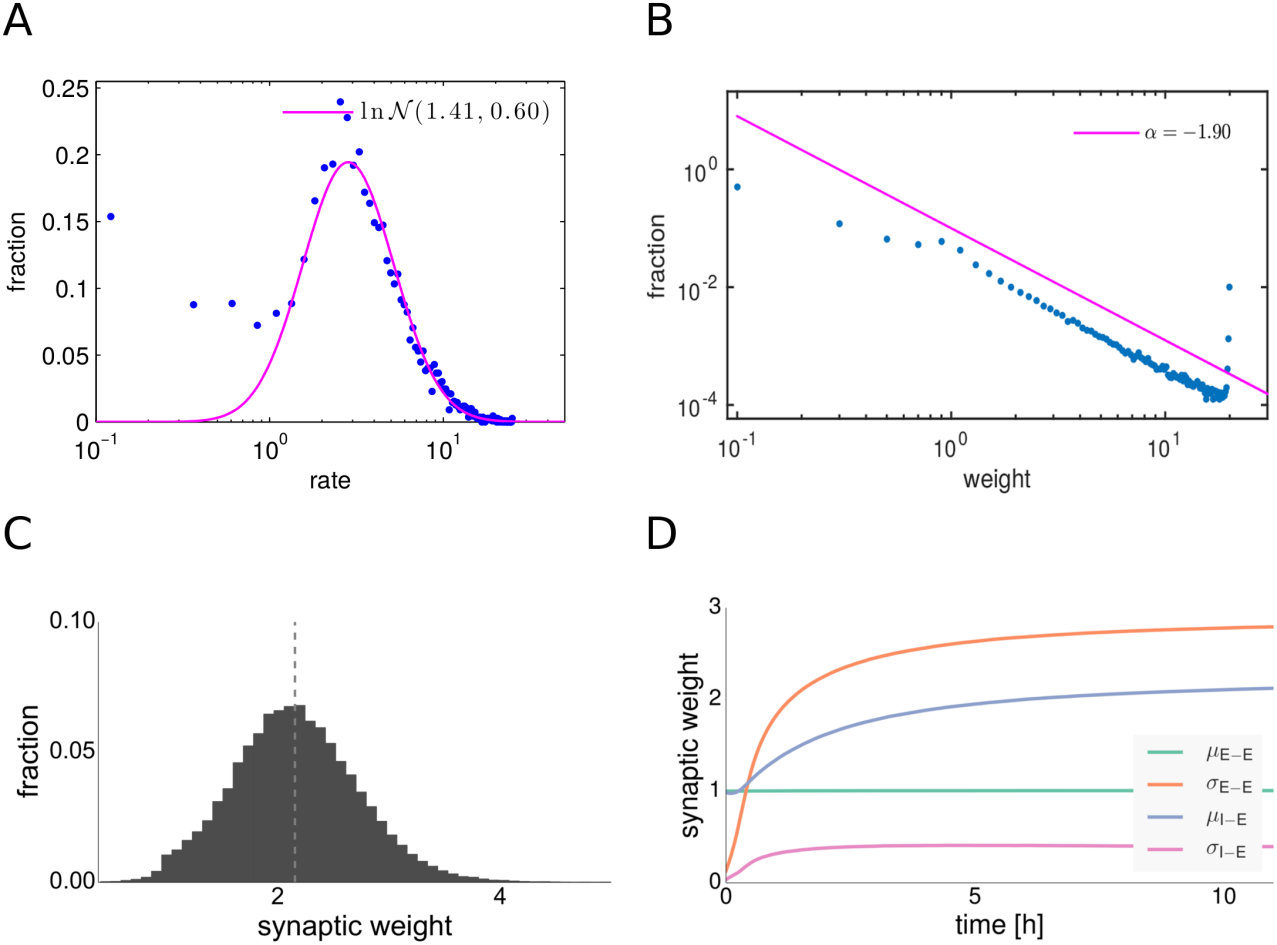
Distributions of firing rates and synaptic weights after synaptic plasticity. **A**: Distribution of firing rates of excitatory neurons. The magenta line shows a log-normal fit. **B**: Distribution of excitatory-excitatory synaptic weights. The magenta line shows a power-law fit to the middle part of the distribution. **C**: Distribution of inhibitory-excitatory synaptic weights. The dashed line indicates the mean. **D**: Evolution of excitatory-excitatory and inhibitory-excitatory weight means and variances.

After a transient phase, the weight distributions of the dynamic E-E and I-E synaptic connections have settled to their new stable shapes (see Fig 1). The mean synaptic strength of E-E connections is kept fixed at a value of 1 by the synaptic scaling rule, but the variance grows rapidly (see Fig 1, 1D). We observed that E-E connections undergo a symmetry breaking and that we obtain a long-tailed distribution of synaptic weights after convergence (see Fig 1, 1B). The EPSP distributions found in cortical and hippocampal networks of excitatory neurons are typically long-tailed [5–7]. Such types of weight distributions can lead to optimal enhancement of the responses of individual neurons to input and are thus beneficial for information transmission at strong synapses, as was shown recently [4, 5, 24]. We used a maximum likelihood estimator for the exponent to fit a power-law distribution with a cutoff into the middle part of the synaptic weights distribution, omitting the strongest 5% of the excitatory synaptic connections. Our fitting procedure was modified from [64]. We found a power-law exponent *α* = −1.92 and an upper cutoff *x*_min_ = 0.205, Fig 1B. Although visually the fit does look good, with the amount of data we produced, it is possible to reject the power-law hypothesis [64]. However, it is clear that the truncated distribution has a long-tail behavior and that the probability decays approximately as a power-law.

We obtained similar results when we exchanged the additive STDP rule with a partly or fully multiplicative one (see Section Different STDP rules).

The weights of the I-E connections evolve to a near Gaussian form. This is due to the fact that inhibitory STDP is subject to negative feedback and thus yields unimodal distributions of synaptic strengths even in the case of a purely additive plasticity rule [54].

In addition to expressing long-tailed weight distributions after convergence, the network rests in the AI regime and expresses approximately log-normal firing rate distributions throughout the transient state (see Fig 1A). We observe many cells firing at very low rates close to 0 Hz and only a few cells firing at rates up to 30 Hz, a property in line with experimental data obtained during spontaneous cortical activity *in vivo* [10] and ubiquitous in brain networks [12].

While log-normal rates are known to be a general and robust property of random balanced state networks with homogeneous weights [23], the combination of both long-tailed distributions of firing rates and synaptic weights is not a straightforward property [2].

### Driver neurons

**Fig 2:**
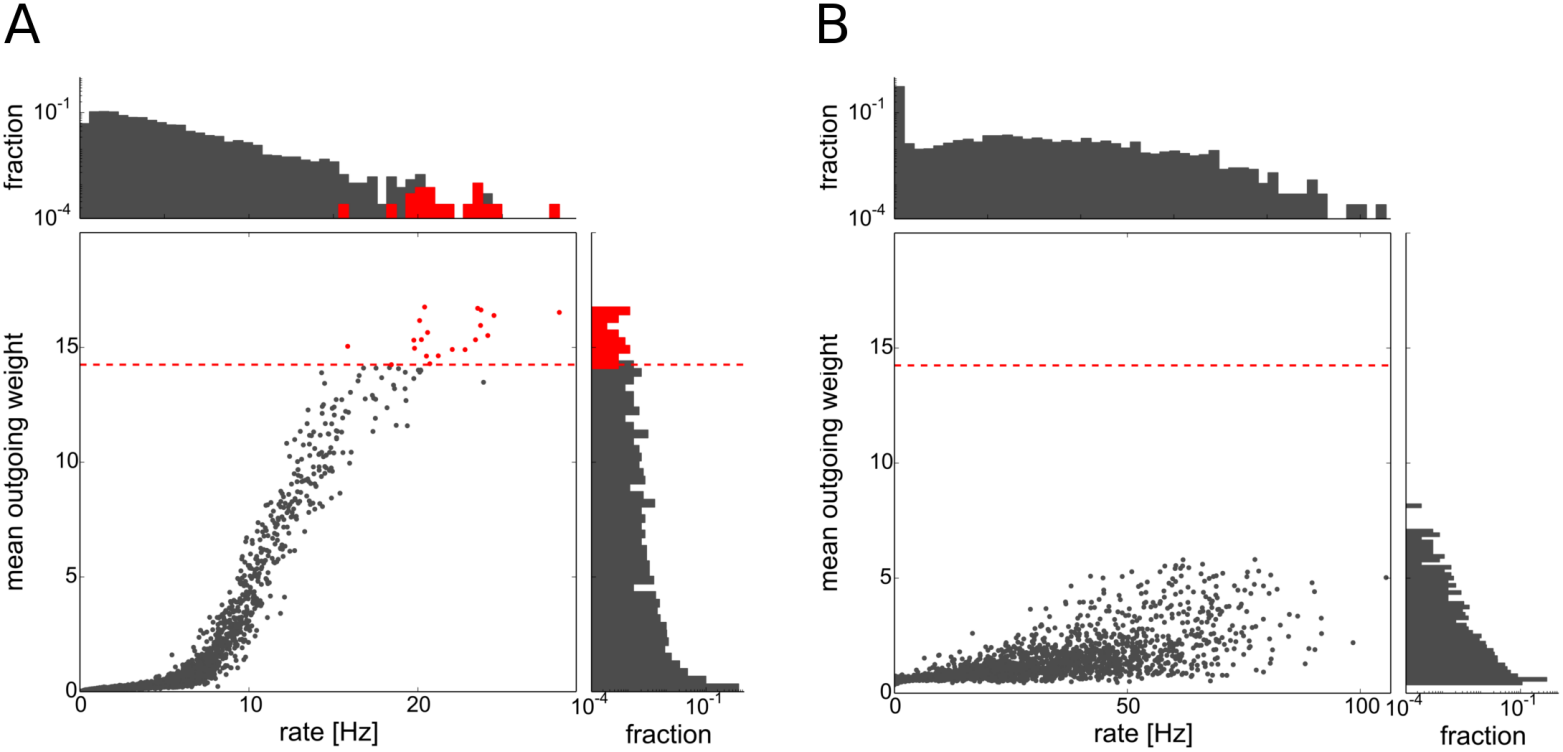
Firing rates and mean outgoing weights of excitatory cells. Each dot represents one excitatory cell. Driver cells are shown in red, others in gray. Dashed red line marks threshold on the mean outgoing weight for the driver cell property. Histograms on the top and the right side show the distribution of firing rates and mean outgoing excitatory weights, respectively. **A**: network including inhibitory STDP, **B**: Network without inhibitory STDP.

Plasticity in the network leads to the development of few exceptionally strong excitatory synapses (see Fig 1B). Interestingly, many of these synapses are found on excitatory neurons which have predominantly strong outgoing connections and which fire at higher than average rates. As we will show in the following, the elevated firing rates are in fact causal for the emergence of their strong outgoing weights (see Section Emergence of driver neurons). We call these neurons *driver cells* (or *driver neurons*) and characterize them by distributions of outgoing excitatory synaptic weights with a high mean value (see Fig 2A).

To define the group of driver neurons we take the top 0.5 percent of the excitatory cells with the largest mean outgoing synaptic strength (see Fig 2A). As our network consists of 4000 neurons, this amounts to 20 driver cells in the network. As the distribution of mean outgoing weights in the network is unimodal, there is no clear-cut threshold separating any group of cells from the rest of the population in this distribution. The particular choice of the threshold results in a very strong dynamical impact of the driver neurons as discussed in section Dynamical impact of driver neurons.

Another possibility to define driver neurons would be to use a threshold located at three standard deviations above the mean. In this way we select neurons that have much stronger outgoing weights than expected under the hypothesis of normally distributed mean outgoing strengths. In contrast to the case of a normal distribution where this choice will result in an expected 0.23% of all cells, we classify 3% of the population as driver neurons using the aforementioned criterion. Using this choice, there is also a detectable dynamical difference between the driver neuron group and the rest of the network, though not as strong as for the 0.5% threshold (see Section 9 in S1 Text).

**Fig 3:**
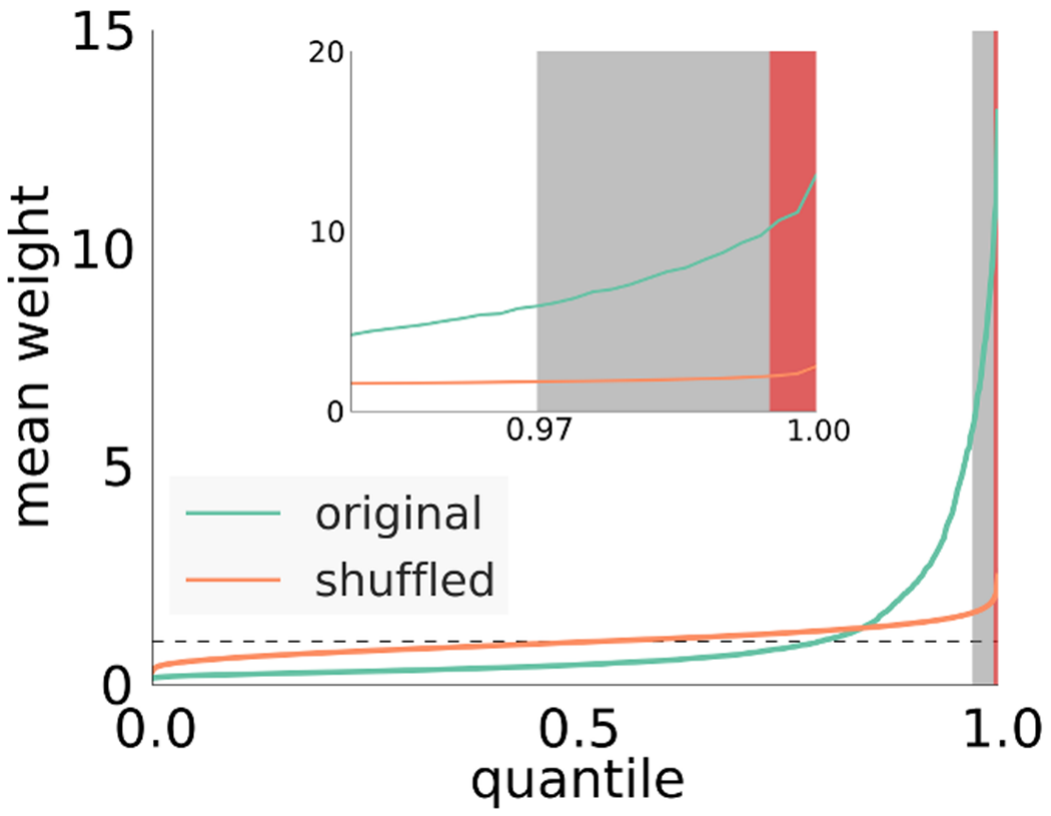
Quantiles of mean outgoing weight distribution of excitatory cells. Green curve obtained from the developed network. Orange curve obtained by shuffling weights at the existing connections, dashed line: the average mean outgoing weight. Shaded areas: Red marks driver cells as they are defined in the manuscript at the 99,5% percentile, gray marks values above three standard deviations from the mean (at the 97% percentile).

The clustering of strong outgoing synapses in the network is shown in Fig 3 (see also Fig U, top left in S1 Text). Here, we plotted the quantiles of the distribution of the mean synaptic strength per neuron in the original network. For comparison we also plotted quantiles of surrogate data obtained by shuffling the synaptic weights among all excitatory synapses. This operation destroys all correlations in synaptic weights introduced by the plasticity mechanisms, while leaving network topology and the overall distribution of synaptic strengths unchanged. As we can see in Fig 3, for the shuffled networks (we show the mean and the standard deviation of 100 shufflings, standard deviation very small and almost invisible in the plot), most cells have a mean outgoing weight of around 1, the mean excitatory weight in the network.

The self-organization of driver cells is due to a delicate interplay of network dynamics and synaptic plasticity. For example, driver neurons are much less pronounced in a model network where we do not include inhibitory STDP (see Fig 2B). We will describe the process of their emergence in more detail in the following.

### Emergence of driver neurons

Looking at driver cells in the equilibrium network and at the same cells early in the network evolution (that we call *future driver cells*), we found that they fire at rates much higher than the network average (network average approximately 5 Hz, driver group average approximately 25 Hz, see Fig 2A). In the following, we will show that this is the main reason for the emergence of their strong outgoing synaptic connections:

STDP dynamics of the excitatory synaptic weights in our network can be seen as a random walk on the closed interval [0, *w*_max_]. Here, the probability to increase the weight grows with increasing synaptic weight, and for weights above a certain threshold the average impact on synaptic weight of each presynaptic spike is positive. In a model without homeostatic plasticity we observed that once a synaptic weight reaches this threshold it converges to its maximum with high probability, with a velocity proportional to the firing rate of the neuron. As we included a postsynaptic homeostatic plasticity rule in our model at E-E synapses that constrains the total sum of weights onto each neuron, this led to a competition of all excitatory synapses converging onto a given excitatory postsynaptic cell over a limited pool of total synaptic efficacy.

**Fig 4:**
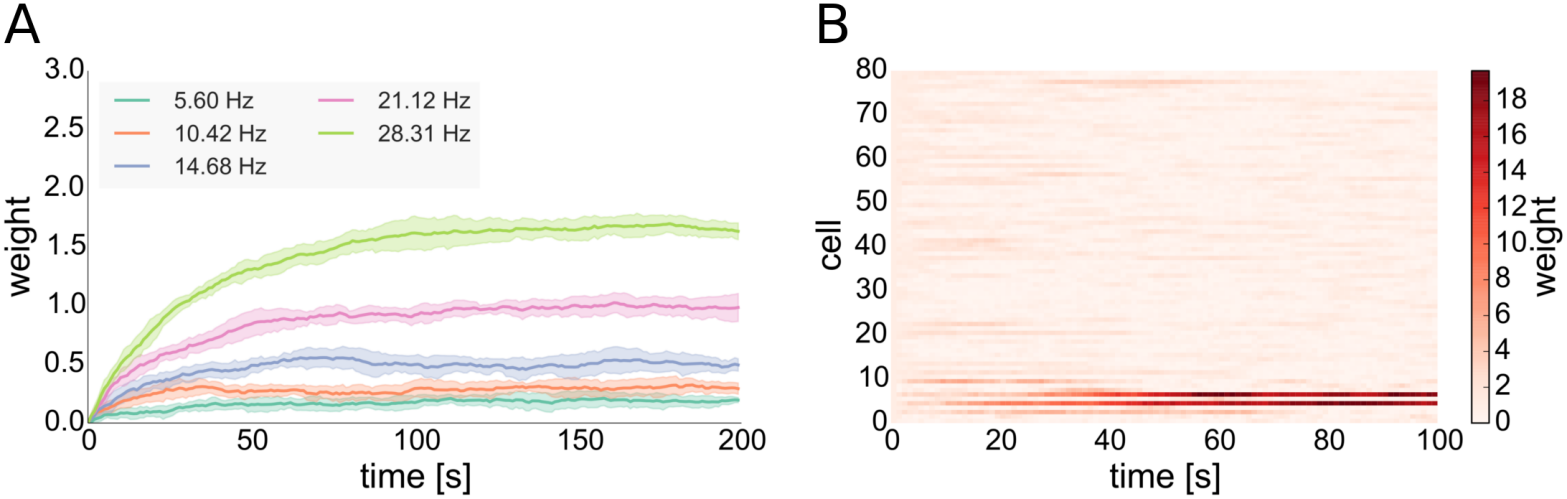
Weight dynamics of inhibitory STDP and of the interplay of excitatory STDP with synaptic scaling. **A**: Evolution of inhibitory synaptic weights of synapses converging onto postsynaptic cells with different rates. The presynaptic cell fires at a rate of around 5 Hz. Solid lines show means of 5 trials, shaded areas standard deviations. **B**: Evolution of excitatory synaptic weights of 80 STDP synapses converging onto one postsynaptic cell with synaptic scaling active at the postsynaptic site implemented in the form of a weight normalization, one trial. All cells fire at rates of around 5 Hz except the first 10 presynaptic cells shown in the bottom rows that fire at around 25 Hz.

One characteristic of many STDP rules as well as the one that we include in our model is that synapses connecting a highly active presynaptic cell with a less active postsynaptic one (in terms of their mean firing rates) tend to undergo LTP [65, 66]. Thus, outgoing synapses of driver cells that fire faster than the average cell have a higher probability to undergo LTP. Synapses from future driver cells are the ones to predominantly win that competition over available synaptic efficacy, diminishing the influence of other cells (see Fig 4B and Section 4 in S1 Text). This ultimately allows driver cells to emerge and to have strong influence on their postsynaptic networks. For our model we support this by analytical considerations (see Methods).

We observe that the higher firing rates of (future) driver cells is due to reduced inhibitory currents those cells receive (see Fig C, left in S1 Text). Currents in our model are influenced by two variables, synaptic weights and presynaptic firing rates. We find that the reduced inhibitory currents that drivers receive are a result of two separate effects of local network topology: First of all, driver cells have a lower than average number of converging inhibitory synapses. Secondly, inhibitory cells which are presynaptic to driver cells have on average a higher number of converging inhibitory synapses than randomly selected inhibitory cells of the network. The latter results in lower than average firing rates of the inhibitory cells presynaptic to driver cells (see Fig D in S1 Text) and the combination of these two effects leads to a permanently reduced inhibitory drive to driver cells. As the included inhibitory STDP rule is subject to a form of self-regulatory dynamics with negative feed-back that becomes stronger with increasing synaptic weight [54], inhibitory plasticity cannot fully compensate this reduced inhibitory drive to driver cells by increasing inhibitory weights: On the one hand, inhibitory STDP in our model tries to compensate the high rate of the under-inhibited neurons by increasing the converging inhibitory weights to those cells. On the other hand, each inhibitory presynaptic spike delays the postsynaptic spike for an increasing period of time with increasing inhibitory synaptic weight, thus decreasing the positive contribution of the STDP rule. This results in a situation in which the converging inhibitory weights become stationary at a value below the maximal inhibitory weight, even in the case of high postsynaptic firing rates as seen in the case of driver neurons (see Fig 4).

Altogether, we thus find that driver cells in our model are mainly determined by (local) network topology and that their emergence is due to an interplay of all three plasticity rules active in the network. In order to test the sensitivity of the observed effects to changes in the network size, we simulate networks of 10,000 and 20,000 cells and find that also in these we obtain qualitatively similar results (see Section 10 in S1 Text).

### Dynamical impact of driver neurons

In the following, we will investigate dynamical and topological properties of the group of driver cells and compare them to randomly sampled groups of non-driver cells of the same size.

One question we wanted to answer is whether the emergence of driver neurons influences the dynamics of the network. To answer this question, we forced both the group of driver cells and a group of randomly selected non-driver cells to fire two consecutive spikes. We observed the network response in both cases. We achieved this by providing two brief pulses lasting 0.5 ms of a very strong constant current input to the group of stimulated cells. Those two pulses were separated by a delay of 2 ms to allow all cells to leave their refractory periods after emitting the first spike. We furthermore only considered cases in which none of the stimulated cells was refractory prior to stimulation so that all cells of the stimulated groups fired exactly two spikes within 2+*ϵ* ms, where *ϵ* ≪ 1 ms is dependent on the membrane potential of the cell prior to the stimulation.

**Fig 5:**
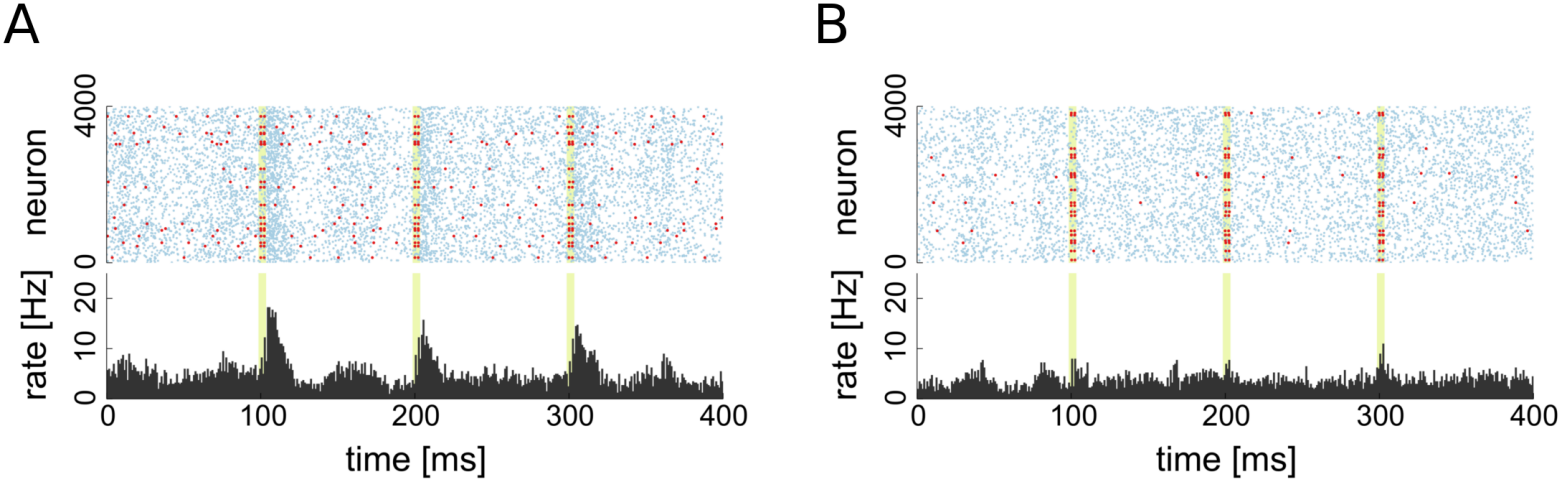
Raster plots of excitatory population activity during stimulation. **A**: Stimulation of 20 driver cells. **B**: Stimulation of 20 random non-driver cells. Times of stimulation are highlighted in light green, red dots indicate spikes of stimulated group, blue dots indicate spikes of non-stimulated neurons. The bottom of each figure shows the PSTH of the excitatory population.

We chose this stimulation protocol to imitate a bursting activity in the chosen subpopulation. To probe a baseline response of the network, we stimulated the same number of randomly selected non-driver cells with the same protocol. In the latter case, the network firing rate rises shortly due to the induced simultaneous firing of the stimulated neurons, but there are no lasting effects on network dynamics (see Fig 5B). On the other hand, the stimulation of the driver cell group results in a prolonged elevation of the firing rate similar to a population spike (see Fig 5A).

**Fig 6:**
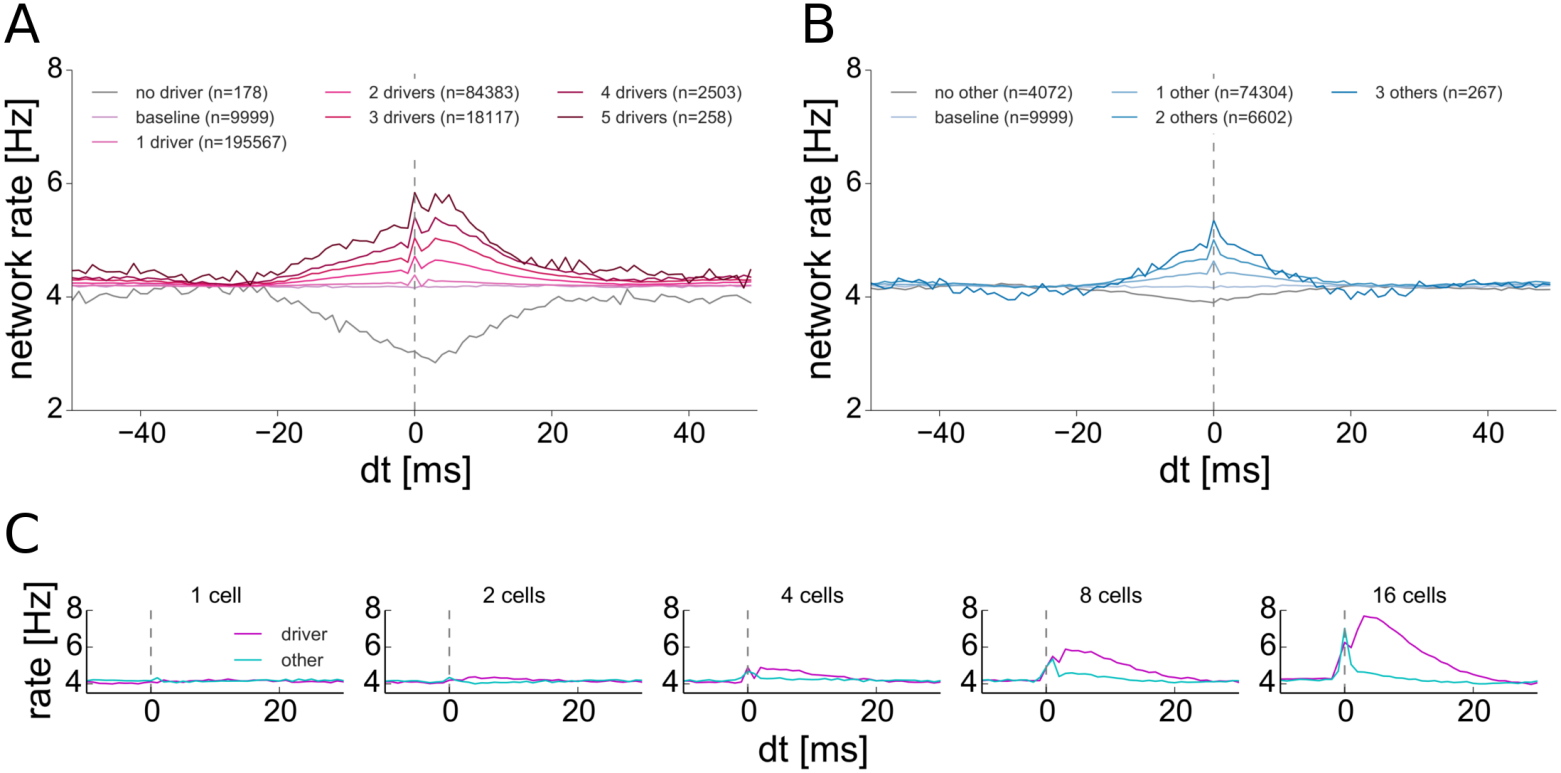
Synchrony triggered averages for driver neurons and group of 20 randomly selected non-driver neurons. **A**: STA for driver cells firing coincidently within 1 ms. **B**: STA for randomly sampled cells firing coincidently within 1 ms. **C**: STA for stimulated groups of 1, 2, 4, 8 and 16 cells. On all plots traces are averages of the excitatory population firing rate and a vertical dashed line marks the time bin of synchronous spiking. In C, magenta line: coincident spiking of driver cells, cyan line: random cells.

To get a more precise picture, we observed the network dynamics subject to the condition that a number of driver neurons spontaneously fire in a synchronous way. We considered events in which a certain fraction of the driver cells all fire within a 1 ms time bin and average the excitatory population rates before and after this event, obtaining a synchrony triggered average (STA) curve (see Fig 6A). For comparison, we sampled a random group of non-driver cells of the same size and considered coincident spikes from its members (see Fig 6B). The STA curves for the random group are symmetric around the moment of synchronization. This indicates that the probability of finding a given number of neurons from this group firing coincidently within 1 ms is higher when the network rate is higher than usual, but this event has no effect on network dynamics. On the contrary, two synchronous spikes from the driver group are sufficient to result in a detectable and causal elevation of the population firing rate. This effect becomes more pronounced for larger groups of driver cells firing synchronously (see Fig 6A). Choosing the top 3% of cells with the highest mean outgoing weights as drivers predictably diminishes the absolute impact of synchronous driver spiking on network dynamics, but qualitatively the results remain unchanged (see Section 9 in S1 Text).

We also performed the same averaging in a setting in which a selected number of driver and random non-driver neurons are forced to spike coincidently by providing a brief stimulation with a strong constant current input (see Fig 6C). The response of the network to the additional synchronous spiking of driver neurons is similar to the case of spontaneous synchrony. A prolonged phase of elevated activity is observed and the effect grows with the number of activated neurons. On the other hand, even after a stimulation of many non-driver neurons, activity rapidly returns to the base-line level and no prolonged change in the network dynamics can be observed.

A recent series of experimental studies performed *in vitro* [18–20] in dissociated hippocampal and cortical cell cultures reported the existence of certain special neurons termed leader neurons. They were characterized by their firing activity stably preceding population bursts in the culture. Leader neurons were found in a wide variety of dissociated cultures obtained from both hippocampal and cortical cells from embryonic, newborn (< 24h) and juvenile (P16–17) rats [18] and under a wide variety of feeding protocols. It was found that synchronized firing of leader neurons increases the probability of the initiation of a population burst above chance level [18]. Furthermore, recent experimental work [20] shows that leader neurons do not just passively precede bursting activity in the cultures, but can actively trigger it.

Whereas our model network stays in the asynchronous irregular activity regime that differs greatly from the synchronized bursting behavior of the hippocampal cultures, there are still periods of elevated activity, and driver neurons fire preceding them and can cause such events if many drivers are triggered to fire simultaneously, a property shared with leader neurons. There are further properties that the driver neurons in our model share with leader neurons, for example the tendency to form functional subnetworks [18, 19] (see Section Stability and topology of the driver neuron subnetwork). Moreover, it was shown [16–18] for leader neurons that the property of early spiking during population bursts is very likely to be caused by synaptic input (reduced inhibition or increased excitation, or a combination of both) to those cells and not intrinsic cell properties (e.g. a reduced firing threshold), a finding we also made for the driver neurons in our model. It is an interesting open question to elucidate what will happen if the network is set to stay in the bursting regime throughout its development, that we consider for a follow up publication.

Moreover, a recent experimental study [3] assesses the existence of subnetworks of highly active excitatory cells in the somatosensory cortex of juvenile mice, expressing both characteristics of leader neurons and driver neurons and thus giving further experimental support for a unification of the two concepts.

### Stability and topology of the driver neuron subnetwork

In earlier studies of balanced state networks with a power-law or multiplicative STDP rule acting at excitatory-excitatory connections [30, 31], strong synapses arising due to a temporary symmetry breaking were not stable and disappeared after some time. In our model the strong synapses diverging from the driver neurons remain strong over long periods of time. Consequently, the property of belonging to the group of driver cells is stable over long periods of network time. This is due to the fact that in our model the property of becoming a driver cell is mainly determined by local network topology. It is an interesting question what will happen in much larger networks. To test for the sensitivity of the results to network size, we simulated networks of 10,000 and 20,000 neurons. We found that increasing network size in the considered ranges did not significantly change the shape of the converged weight distributions or the clustering of strong outgoing weights (see Section 10 in S1 Text).

We furthermore observed that driver cells form “rich club” subnetworks in which most synapses are strong. This can be explained by the fact that driver cells emerge in waves, recruiting cells from their postsynaptic networks. As the outgoing synaptic connections of future driver cells which fire at much higher than average rates undergo LTP, this leads to elevated excitatory currents in the cells postsynaptic to driver cells and to an increase in their firing rates. This, in turn, increases the chances of the postsynaptic cells of also becoming driver cells. This iterative process terminates at some point as the total available synaptic weight in the network is limited by a homeostatic rule and the fact that inhibitory STDP up-regulates converging inhibitory weights onto cells with elevated firing rates.

To illustrate the effect, we simulated 1000 different networks and looked at the driver cells that emerged in those networks and their subnetworks. To measure the connectedness of the subgroups we studied the number of synaptic links within two groups of *n* = 20 neurons each, the group of driver neurons and a group of randomly sampled non-driver neurons. We found that the driver cell group has a significantly higher number of synaptic connections *C*_driver_ compared to the number of synaptic connections *C*_rand_ in the random group. The random group on average expressed *C*_rand_ = 7.35 ± 3.30 (mean ± standard deviation) synaptic connections in their subnetwork. Not surprisingly, this is very close to the number of expected synaptic connections in a random network of 20 nodes with connection probability *p* = 0.02 which is *C*_0_ = 20⋅19⋅0.02 = 7.6. In contrast, we found on average *C*_driver_ = 12.14 ± 2.65 predominantly strong synaptic connections in the driver neurons subnetwork, an almost twofold increase compared to the random group. Similar relations were found in recent experimental studies of developing cortical networks. Stable subnetworks of more active cells were found to express a higher amount of connectedness [3]. Moreover, a tendency to higher mean EPSP amplitudes with lower variances within highly connected subnetworks was found [7].

**Fig 7:**
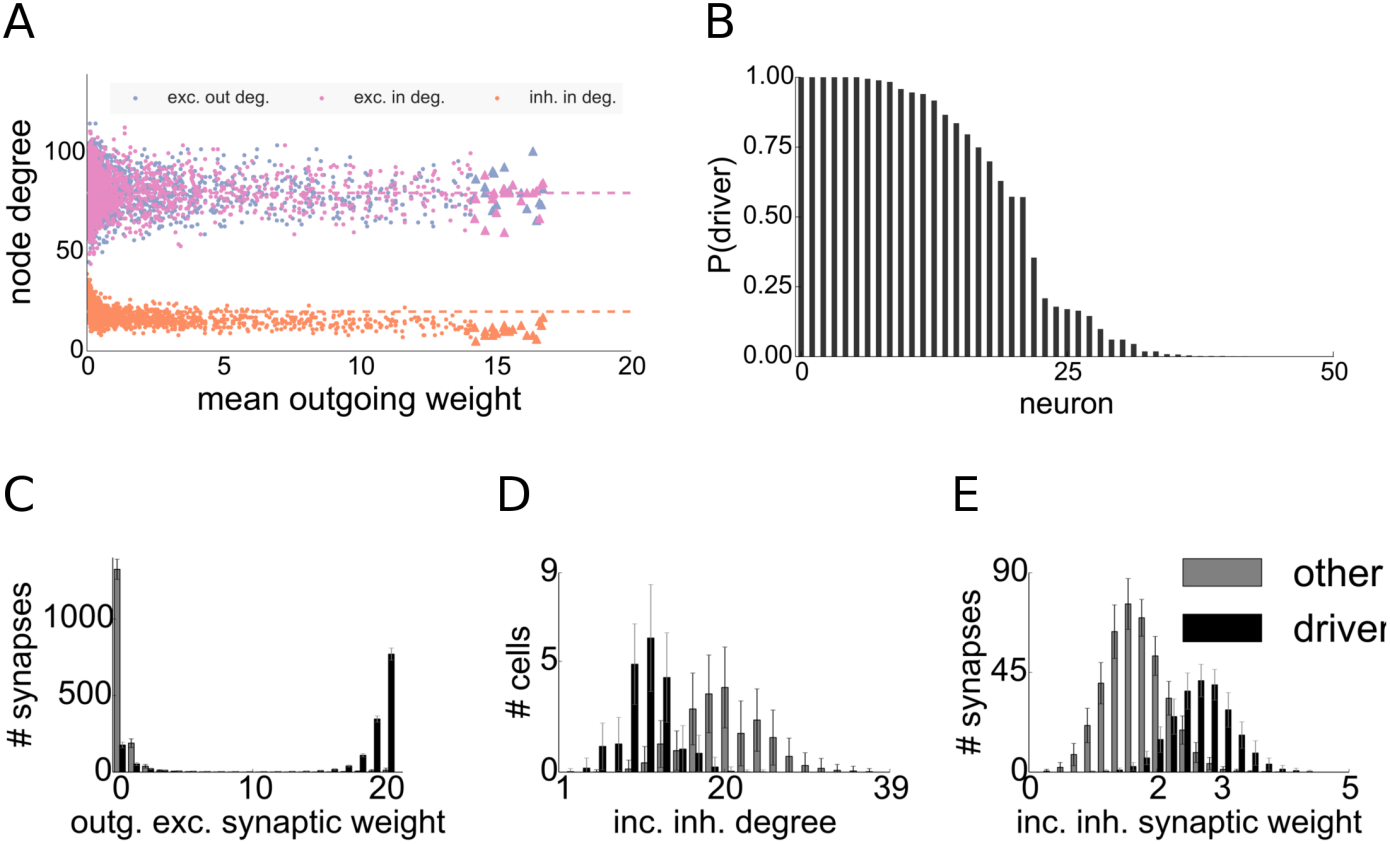
Statistics and connectivity of driver cells. **A**: Scatter plot of the mean outgoing synaptic weight after synaptic plasticity versus node degrees of excitatory cells for one network realization. Dashed lines denote corresponding means. **B**: Probabilities of excitatory cells to belong to the driver group, computed by sampling *N* = 1000 network realizations with fixed topology but different input. Descendingly sorted by probability. **C-E**: Statistics of connectivity of driver cells and a group of 20 randomly sampled non-driver cells. **C**: Distributions of the strengths of outgoing synapses, **D**: Inhibitory in-degree, **E**: Efficacies of incoming inhibitory synapses. Bars denote average over 1000 trials, whiskers indicate standard deviation.

The question remains whether there are other topological properties that distinguish driver neurons from the rest of the population, apart from the reduced inhibitory in-degrees. To answer this question we measured both excitatory and inhibitory in- and out-degrees throughout 1000 different networks. We found that the number of incoming and outgoing excitatory synaptic connections does not distinguish driver neurons from the rest of the network in our model, as we can already see in the example network (see Fig 7A).

To asses the observed difference in inhibitory in-degrees more clearly, for each network simulation we extracted the driver group after convergence of the synaptic weights and took one random group of non-driver neurons of the same size. As expected from the definition, the distribution of outgoing excitatory synaptic weights allows to distinguish the two groups of neurons dramatically (see Fig 7C). The distributions of incoming inhibitory degrees are also easily separable across the two groups (see Fig 7D). To compensate the smaller incoming inhibitory degree, inhibitory STDP up-regulates inhibitory weights converging onto driver cells such that they receive on average much stronger incoming inhibitory weights (see Fig 7E). But as discussed earlier, they none the less receive reduced inhibitory currents when compared to the network average (see Section Inhibitory STDP).

To verify our hypothesis that driver cells in our model are mainly distinguished by properties of local network topology, we performed simulations of 1000 networks with varying initial conditions but a fixed network topology. To induce statistical fluctuations, we stimulated the networks with Poisson noise and then looked at the probability (assessed via the relative frequency) of each cell to belong to the driver group after the synaptic weights have converged. We found that the driver neuron population remained mainly unchanged independently of initial conditions and that roughly 30 different neurons were found in the driver cell group across all trials (see Fig 7B). The source of the variation between the outcomes is in the random initialization and input fluctuations. Altogether, the property of belonging to the driver group is thus not solely but mainly dependent on network topology.

Another interesting question is how large the variation in local network connectivity has to be in order to allow for the emergence of driver cells. To answer this question, we simulated a fully homogeneous network in which all cells have the same in-degree of both excitatory and inhibitory connections. In this case the weight distributions almost remained delta peaks, i.e. each synaptic weight stayed *w* ≈ 1 even subject to plasticity and no driver cells emerged (see Fig I, left in S1 Text).

Interestingly, already a slight amount of under-inhibition suffices to allow for the emergence of driver neurons. We demonstrated this by taking a random group of 50 cells in the fully homogeneous network and selectively pruned 10% of the inhibitory synapses converging onto each cell of the group. We found that already this small change in homogeneity suffices to allow the group to become driver cells (see Fig I, right in S1 Text).

### Inhibitory STDP

What is the role of inhibitory plasticity in our model? Similar to recent theoretical studies [53–56], inhibitory STDP plays a stabilizing role in our network setup. Yet, inhibitory plasticity furthermore plays a crucial role in the emergence of driver neurons, as we will see in the following.

In order to assess the effect of inhibitory STDP more closely, we simulated networks with static I-E synaptic connections of constant weight (see Section 7 in S1 Text). The magnitude of the constant inhibitory weights was selected to be equal to the mean of the equilibrium distribution of inhibitory weights in the same network with inhibitory STDP. Without inhibitory STDP, the network still exhibits mostly asynchronous irregular activity, but with a higher amount of oscillations and with some cells expressing firing rates of up to 100 Hz (see Fig F in S1 Text). Also, the excitatory population firing rate almost triples (with a mean rate of around 15 Hz) in this case, whereas the inhibitory population firing rate does not increase so drastically. The excitatory weight distribution is also similar to the case of a network including inhibitory STDP (see Fig G in S1 Text), but outgoing strong weights cluster much less on the subset of neurons that constitutes the driver cell group in the former case (see Fig 2B and Fig U in S1 Text), making their dynamical impact on network dynamics much smaller (see Fig H in S1 Text). We furthermore found that this result does not depend on the actual value of the fixed inhibitory weight, but that it can be observed even for very weak or strong fixed inhibitory connections. How can this be explained?

The difference between plastic and static inhibitory connections lies in the selective nature of the Hebbian inhibitory STDP rule [44]. Namely that it increases the synaptic weights converging on cells with higher firing rates more strongly than the ones converging onto cells with lower firing rates (see Fig 4A). This is the mechanism underlying its stabilizing property. Without inhibitory STDP, slightly under-inhibited neurons (that could develop into driver neurons in the fully plastic network) fire with higher rates and by this alone increase the firing rate of their postsynaptic partners. This happens on time-scales that are much shorter than the ones of synaptic plasticity. Thus, neurons that are post-synaptic to under-inhibited cells attain higher firing rates and start competing with their presynaptic partners over the available pools of postsynaptic weights limited by the synaptic scaling rule (see Fig 4B). This process, although also yielding long-tailed distributions, prevents the strong clustering of strong outgoing synapses on single cells (see Fig 2B and Section 13 in S1 Text). The cells with the highest mean outgoing weight also have an impact on network dynamics in this case, but this is much less pronounced than in the case of a network including inhibitory STDP (see Fig H in S1 Text).

### Generality of the model

Do the results depend on parameter tuning? Do the the results generalize to learning rules other than additive STDP? For this we will refer to the previously described model with additive excitatory and inhibitory STDP rules and a synaptic normalization at excitatory synapses as the *base model* and study variations of it.

#### STDP learning rates

First of all, we found that in the base model results do not qualitatively depend on a tight tuning of the parameters of the plasticity rules employed. We verified this by simultaneously varying the learning rates of the excitatory and inhibitory STDP rules while assessing both network dynamics and the dynamics of synaptic weights during convergence to their equilibrium distribution. We found little difference both in network dynamics and stable weight distributions under co-variation of the learning rates of the two STDP rules by one order of magnitude into each direction from its initial value (see Methods). Yet, the relative magnitudes of excitatory and inhibitory STDP learning rates do influence both network and weight dynamics: If the quotient of excitatory to inhibitory STDP learning rate becomes too large, positive feedback results in an over-excitation of the network, leading to pathologically high firing rates and strongly bimodal weight distributions.

#### Different STDP rules

Apart from the additive STDP rule [26] that we included in the base model, also classes of partly multiplicative (characterized by additive potentiation and multiplicative depression [48]) and fully multiplicative STDP rules (where both potentiation and depression are multiplicative) were proposed and studied previously [51]. How are the results influenced by different choices for the STDP rules?

Exchanging the additive STDP rule at E-E synapses with either a partly or fully multiplicative one resulted in a network that still expressed long-tailed distributions of firing rates and long-tailed distributions of the excitatory weights (see Section 12.1, Section 12.2 in S1 Text). In these settings, the EPSP distribution was much less widespread than the one in the base model due to the fact that in contrast to a purely additive rule, stronger synapses are subject to much stronger LTD in these cases. This results in a less pronounced dynamical impact of cell spiking on their postsynaptic network dynamics compared to the base model, even for the cells with strongest outgoing mean weights. Yet, we still observed a clustering of the strongest outgoing synapses on cells with higher firing rates, even to a greater degree than in the case of an additive STDP rule, see Section 13 in S1 Text. So, although in this setup the dynamical effect of the cells with the highest outgoing mean weight on network dynamics is much less pronounced due to the smaller absolute synaptic weights occurring compared to the base model, driver neurons still emerge.

In contrast to excitatory STDP rules, there is a much greater variety of inhibitory STDP windows [43, 55]. We used a Hebbian rule as inspired by measurements in entorhinal cortex [44] in the base model, as was done in other modeling studies [54, 56]. If we exchange the inhibitory STDP rule of the base model with the one proposed in the modeling study [53], we find that neither the firing rate nor the weight distributions become long-tailed. This is due to the strong rate-normalizing nature of this rule [53] that also does not allow for driver neurons to emerge.

#### Different forms of homeostatic plasticity

Unlike the synaptic normalization [59] we included in the base model, the homeostatic plasticity rule described in [28] acts on slower time scales. We found that replacing the synaptic normalization rule with a homeostatic plasticity rule that acts on timescales slower than STDP (see Methods) results in a network that expresses similar weight and firing rate distributions as the base model (see Section 12.3 in S1 Text). As in the base model, we observe a clustering of the strongest outgoing synapses on driver cells (see Section 13 in S1 Text).

#### Network topology

**Fig 8:**
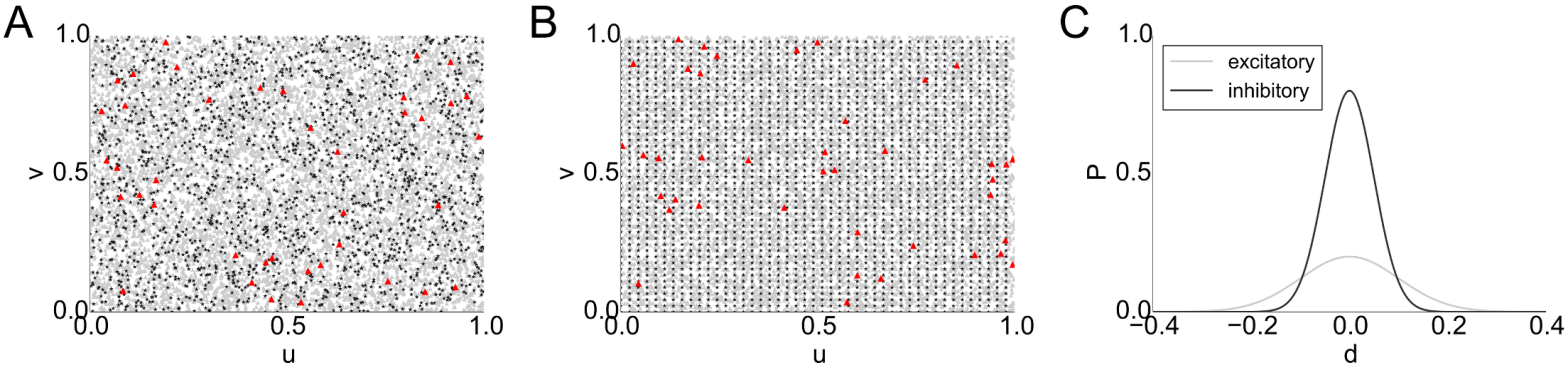
Topographic network with local connectivity on a torus. **A, B**: Positions (*u*, *v*) of 10,000 cells on a torus (left and right, top and bottom edges are identified), excitatory cells shown as gray triangles, inhibitory cells as black stars, driver cells in red. **A**: Uniformly distributed excitatory and inhibitory cells. **B**: Inhibitory cells grid-aligned, excitatory cells uniformly distributed. **C**: Local connectivity profiles of excitatory and inhibitory cells.

To answer the question whether the results are influenced by non-local aspects of network topology, we also simulated networks with local, distant-depended connectivity profiles that are often taken as models for cortical connectivity [67]. So far, experimental knowledge (reviewed in [68]) as well as modeling approaches (e.g. [67]) for inhibitory to excitatory population connectivity, which plays a major role in our analysis, are rather unspecific. The common agreement is to assume inhibitory connectivity to be local and dense, with very high connection probability to the nearby neurons [69, 70]. To incorporate these constraints, we thus considered locally-connected topographic networks with neurons positioned on the surface of a torus (which allows us to work without boundary conditions). In accordance with previous studies, excitatory neuron positions were drawn from a uniform distribution, and inhibitory neurons were either placed on a grid (as in [67]) or also drawn uniformly (see Fig 8A, 8B). The networks consisted of 10,000 neurons, and each neuron was connected to its neighbors with a probability depending on the Euclidean distance between the cells. Specifically, the connection probability *P*(*a*, *b*) between two cells *a* and *b* was chosen to be $P(a,b)=\text{exp}(-\epsilon \frac{\text{d}{\left(a,b\right)}^{2}}{2\sigma^{2}})$, where d(*a*, *b*) denotes the Euclidean distance between *a* and *b* and *σ*, *ϵ* are shape parameters of the connection profile. To account for the parameters found in experimental studies, we parametrized the torus as [0, 1]^2^ and chose connection profiles with *ϵ* = 0.2, *σ* = 0.1 for excitatory cells and *ϵ* = 0.8, *σ* = 0.05 for inhibitory cells (see Fig 8C). We found that the networks behave qualitatively like the previously considered random networks: we also obtained long-tailed firing rate and weight distributions (see Section 11 in S1 Text). We furthermore found driver cells to be scattered uniformly across the population (see Fig 8A, 8B). As in the base model, sub-networks of driver cells display higher connectivity than groups of randomly sampled non-driver cells. To assess this, we simulated 10 different networks on the torus and found 32.11 ± 6.45 (mean ± standard deviation) synaptic connections between the 40 driver cells, whereas the number of connections in a randomly sampled subgroup of 40 non-driver cells was found to be 18.11 ± 5.47. This is a result of the recruitment process described previously.

Altogether, we can thus say that the phenomena discussed above such as the expression of long-tailed weight distributions and the emergence of driver neurons and subnetworks of such are a generic feature, stable even under alternations of the plasticity rules and their parameters.

## Discussion

We presented here a model of a balanced state network of spiking neurons in which a set of biologically plausible plasticity rules such as STDP and homeostatic plasticity leads to stable effects of self organization.

In contrast to previous studies, structures promoting particular forms of signal propagation appear in a self-organized way in our model rather than being imprinted statically [71], and are stable over long periods of time rather than a transient feature [30]. Furthermore, inhibitory STDP stabilizes network dynamics in our model, keeping it in a biologically plausible regime and it eliminates the problem of runaway excitation and pathological network states upon the repeated synchronous stimulation of a group of neurons faced by some previous models [31].

Starting from a homogeneous, Gaussian or uniform configuration, the network expresses long-tailed distributions of synaptic weights after a transient phase. Synaptic weight distributions were also found to be long-tailed in cortical networks [6, 7, 72, 73] and such distributions were shown to facilitate information processing in spiking networks [5, 24].

Additionally, the network expresses long-tailed distributions of firing rates and a combination of the two properties of having both long-tailed distributions of firing rates and synaptic weights is not straightforward [2]. For example, the weights in the SORN model [33] converge to a long-tailed distribution, but the distribution of firing rates is near-Gaussian. Taken together, both the long-tailed distributions of synaptic weights and firing rates constitute an interesting property of the model, in particular as over the last years increasing evidence surfaced that long-tailed distributions are ubiquitous in biological neural networks and might play an important role in brain functioning [10, 12]. Moreover, the network stably expresses asynchronous irregular spiking activity, a regime that is believed to be a good theoretical fit to cortical activity *in vivo* [34, 35, 37].

A delicate interplay of the excitatory and inhibitory plasticity rules in our model allows a fraction of the excitatory cells that we call *driver neurons* to develop predominantly strong outgoing excitatory synapses. We showed that driver cells have a strong impact on the dynamics of their postsynaptic networks and that by synchronous spiking activity they can even trigger population bursts. Characteristic properties of driver cells were found to be much higher than average firing rates caused by reduced inhibitory currents that they receive (leading to a higher relative excitatory drive) and higher degrees of connectivity in their subnetworks.

As a result, driver neurons express a high degree of “effective embeddedness” [74] within the network and they can help to bridge the gap between single-cell and network activity, a possibly relevant dynamical connection, as *in vivo* even single spikes were shown to matter both on the level of network dynamics [75] and behaviorally [76].

Local network connectivity played a crucial role in the emergence of driver cells, in particular local imbalances in the number of converging excitatory and inhibitory synapses. Unfortunately, not much experimental data are available providing information about the (co-)variance of the number of converging inhibitory and excitatory synapses onto single neurons. The few studies that we could find assessed variations of synapse numbers between 10% and 30% per cell [77, 78], which would be sufficient to allow for the emergence of driver neurons in our model. As from a theoretical point of view the determining factor in the emergence of driver neurons is the quotient of excitatory and inhibitory currents that such cells receive, they could equivalently emerge by an increased excitatory drive, possibly accompanied by reduced inhibition, and we expect to be able to selectively form driver cells and assemblies of driver cells by providing appropriate input patterns to a network. We tested sensitivity of the observed processes of self-organization to changes in network size. For the cases we tested (10,000 and 20,000 neuron networks), we found no big differences in the results depending on network size. We leave a more thorough investigation of this question to a follow-up work.

We expect the emerging strong synapses, driver neurons and subnetworks of driver cells to provide an efficient substrate for the generation of stereotyped recurring patterns of neural activity, in particular when the network is presented a more meaningful (i.e. structured) input. Such patterns are a ubiquitous phenomenon observed across different species and brain regions both *in vitro* and *in vivo* [79–82] and are believed to play an important role for both information transfer and processing in neuronal networks. Moreover, emergent subnetworks of driver neurons can promote synchrony in the network, an aspect of network dynamics that has been shown [83, 84] to play an important role in neuronal interactions and the gating of sensory information.

The observed phenomena of self-organization are not strongly dependent on the initial distributions of synaptic weights, a tuning of the parameter values of the plasticity rules or even the class of learning rules employed. For example, the qualitative results remain unaffected if the time scale of the homeostatic plasticity rule is increased by several orders of magnitude [28], and strong outgoing weights still cluster on highly active cells when exchanging the additive STDP rule at E-E connections with a partly or fully multiplicative one [48, 51]. Therefore, the observations we report here seem to be an emergent generic feature rather than an artifact due to certain parameter choices or even specific learning rules. We furthermore expect phenomenological models of activity-dependent synaptic plasticity that incorporate a rate dependence of LTP [85, 86], as previously observed experimentally [65], to yield qualitatively similar or even more pronounced results regarding the emergence of driver neurons as they favor strong synaptic connections at cells with high firing rates.

The model we present here employs a well-understood network architecture and biologically plausible plasticity rules as building blocks. It is also minimal in the sense that if any one of the plasticity rules is excluded, the qualitative results change and the described features of self-organization, such as the emergence of driver neurons, are not observable. If synaptic scaling for the excitatory connections is excluded, the E-E weight distribution becomes bimodal and the network tends to be in an overly excited, synchronized state. Finally, if inhibitory STDP is excluded, this yields much less prominent driver neurons. In this case, despite the fact that the E-E weight-distribution is long-tailed, clustering of outgoing strong synapses is much weaker due to increased competition between the cells. This is due to the fact that both homeostatic plasticity and inhibitory STDP influence synaptic competition in our model, albeit on different levels. Whereas homeostatic plasticity introduces competition between synapses, inhibitory plasticity decreases it between neurons, allowing driver cells to predominantly form strong outgoing connections by suppressing cells postsynaptic to driver neurons. This is a general requirement for development of driver neurons: there should be competition on the level of single synapses, but not too much competition on the neuronal level, and there should be some amount of inhomogeneity in the network structure to seed the symmetry breaking. Those requirement are fulfilled in different settings, most easily in the network we described in this article that is equipped with additive STDP, inhibitory STDP and synaptic scaling.

Despite its simplicity, our model is supported by a multitude of recent experimental findings. For example, studies investigating the architecture of cortical microcircuits [1, 3, 7] already constitute some experimental verification of certain aspects of our findings. In [1], the excitatory network architecture of the C2 barrel column in juvenile (P18–21) mice was found to express rare large-amplitude EPSPs at excitatory cells, and those were hypothesized to play an important role for the dynamics and information processing in the network by providing a substrate for the emergence of strongly connected functional cell assemblies. In our model, synaptic plasticity leads to a similar situation in which the strong outgoing synapses at driver neurons allow them to take strong influence on both their postsynaptic networks and on the whole network by means of forming strongly connected subnetworks of driver cells. Moreover, the combination of higher degrees of connectivity accompanied by EPSP distributions with higher means as found in the emerging driver subnetworks is in line with experimental data from cortical networks in the somatosensory cortex of juvenile (P14–16) rats where mean EPSP amplitudes were shown [7] to increase with the degree of synaptic connectivity within cell groups.

Another recent experimental study [3] finds a strongly interconnected subnetwork of highly active fosGFP^+^ excitatory neurons in the barrel cortex of juvenile (P13–23) mice that is stable over longer periods of time. Rather than being a cause of cell-intrinsic electrophysiological properties, the elevated firing rates were found to be caused by a combination of decreased inhibitory and increased excitatory input to those cells due to network activity [3], a similar situation as observed for the driver neurons in our model. Moreover, the fosGFP^+^ neurons were found to be more effective at driving recurrent network activity than their fosGFP^−^ partners that are characterized by lower activity. At the same time, fosGFP^+^ cells were shown to be preferentially active in early periods of spontaneous activity, a property shared with leader neurons. A speculative, but intriguing thought is to regard the study [3] as the first experimental investigation of leader neurons in non-dissociated cultures, that, if the hypothesis was true, would provide further experimental evidence that leader neurons possess many characteristic properties (higher firing rates, lower inhibition, ability to drive network activity) of the driver cells in our model.

Moreover, driver cells in our model share many properties with leader neurons that were found in experimental studies of a wide range of different dissociated hippocampal and cortical cultures obtained from embryonic, newborn (<24h) and juvenile (P16–17) rats [16–20], making them a seemingly ubiquitous phenomenon of developing neuronal networks. Yet, we want to stress that leader neurons so far were only investigated in cultures expressing bursty activity with longer intermittent periods of quiescence, whereas our model expresses asynchronous irregular activity. The emerging subnetworks of driver cells in our model show dynamical properties similar to functional assemblies of leader neurons termed “primal circuits” [19] and “burst initiation zones” [17] (in the case of 1D cultures) and are also stable over long periods of time [19]. Specifically, leader neurons were found to have higher than average spiking activity [19] and to form functionally well connected circuits that collectively lead most of the observed network bursts [18, 19], similar to driver neurons in our model. Moreover, recent experimental studies [18, 20] on leader neurons show that they do not just passively lead population spikes but are also able to trigger them, akin to our model in which synchronous spiking activity in the driver subnetwork can trigger network bursts. In particular, subnetworks of leader neurons were hypothesized to provide an explanation of the observed patterns of spontaneous and evoked activity [18] and in our model we find exactly such subnetworks of driver neurons emerging, albeit in a likely much more simplified form than to be expected in biological networks.

Since our model enables the investigation of the interplay between network dynamics and structure, it allows us to make predictions of structural properties of neural networks that were not experimentally investigated so far, as well as raising further questions that could be tested experimentally. Regarding leader neurons, our model predicts those cells to receive reduced inhibition. Apart from the previously discussed experimental support for this prediction, another piece of evidence is given by the finding that burst initiation zones in 1D networks of developing cultures of cortical neurons (that correspond to leader neurons in the 2D case) were found to have an almost 3-fold reduced density of inhibitory neurons compared to adjacent areas [17], an effect similar to the reduced inhibition of driver neurons that we found in our model. Whether the same also holds for leader neurons in 2D cultures remains to be investigated. Another observation following from our model is that decreasing the amount of inhibition present in the network can suppress the emergence of driver neurons. It would be interesting to see how this relates to the experimental findings showing that groups of leader neurons become unstable and express a large turnover in memberships when the culture is subject to a blockage of GABA_A_ receptors [17, 19].

Moreover, a recent study [11] recording *in vivo* from hippocampal neurons in rats finds both a long-tailed distribution of firing rates with the activity of each cells being similar across a multitude of different brain states, and at the same time strong evidence for a long-tailed distribution of synaptic weights, assessed via spike transmission probabilities. This is related to an additional question raised by our model, namely whether further experimental evidence can be found for the existence of cells and (functional) subnetworks of such cells that constitute both the long tail of the distributions of firing rates and synaptic weights. To answer this question, both firing rates and synaptic connectivity patterns of a given neural population have to be known, a challenging and interesting question for future experimental work.

### Conclusion

In this paper we examined the self-organization of inhomogeneous synaptic strengths in balanced networks. Beyond the development of long-tailed weight and rate distributions, we observed a clustering of the strongest outgoing synapses on a few neurons that we call driver neurons. This clustering stays qualitatively the same for different modifications of the STDP rules, homeostatic regulations, and network topology. Our analytic results demonstrate how the network enhances small initial inhomogeneities by a combination of three plasticity rules: excitatory STDP, inhibitory STDP, and homeostatic plasticity. We furthermore showed that inhibitory STDP can serve not only the purpose of circuit stabilization, but also how it might be central for structure formation in networks.

## Methods

We study a classical random, balanced state network of leaky integrate and fire neurons with current-based synapses [34, 35, 71]. We simulated the network using the Brian simulator software [87].

### Neuron model

The sub-threshold membrane potential of each LIF neuron obeys

$$\begin{array}{c} \tau_{\text{m}}\frac{dV}{dt}=-\left(V-E_{\text{L}}\right)+I_{\text{e}}^{\text{syn}}-I_{\text{i}}^{\text{syn}}, \end{array}$$

where *τ*_m_ = 20 ms is the membrane time constant, *E*_L_ = −60 mV denotes a leak term and $I_{\text{e}}^{\text{syn}}$, $I_{\text{i}}^{\text{syn}}$ denote excitatory and inhibitory synaptic currents, respectively. Whenever the membrane potential crosses a spiking threshold *V*_thres_ = −50 mV, an action potential is generated and the membrane potential is reset to the resting potential *V*_reset_ = −60 mV, where it remains clamped for a refractory period *τ*_ref_ = 2 ms.

The excitatory synaptic currents are given by

$$\begin{array}{c} I_{\text{e}}^{\text{syn}}=w_{\text{e}}c_{\text{e}}^{\text{norm}}g_{\text{e}}, \end{array}$$

where *g*_e_ denotes the presynaptic spike train that is convolved with a synaptic kernel function, $c_{\text{e}}^{\text{norm}}=1$ mV is a normalizing factor and *w*_e_ denotes the synaptic weight normalized to values [0, *w* ^max^] with $w_{\text{e}}^{\text{max}}=20$, and an initial weight *w*_e_ = 1.

The negative synaptic current $I_{\text{i}}^{\text{syn}}$ is defined analogously with $c_{\text{i}}^{\text{norm}}=-9$ mV, $w_{\text{i}}^{\text{max}}=5$, and an initial synaptic weight *w*_i_ = 1.

Synaptic connections are current-based with exponential kernel functions $\tau_{\text{e}}\frac{dg_{\text{e}}}{dt}=-g_{\text{e}}$ and $\tau_{\text{i}}\frac{dg_{\text{i}}}{dt}=-g_{\text{i}}$. Here, *τ*_e_ and *τ*_i_ denote excitatory and inhibitory synaptic time constants, respectively. They are chosen as *τ*_e_ = 5 ms and *τ*_i_ = 10 ms, in accordance with fast-acting excitatory and inhibitory neurotransmitters.

Synaptic parameters are chosen so that effective EPSP and IPSP amplitudes are comparable with experimental data [88]. EPSP amplitudes take values between 0 mV and 2.25 mV (corresponding to a synaptic weight of $w_{\text{e}}^{\text{max}}=20$), with 0.16 mV corresponding to an excitatory synapse with weight *w* = 1. IPSP amplitudes take values between 0 mV and −11.23 mV (corresponding to a synaptic weight of $w_{\text{i}}^{\text{max}}=5$), with −2.25 mV corresponding to an inhibitory synapse with weight *w* = 1.

### Network architecture

We consider a random, balanced state network of leaky integrate and fire neurons consisting of *N* = 5000 cells of which 4000 are excitatory (E) and 1000 inhibitory (I). The network is fully recurrent with all four connection types E-E, E-I, I-E and I-I present. The probability of a synaptic connection between any two neurons is *p* = 0.02, a value chosen as a compromise between the higher connection probabilities found for neighboring cortical neurons and the lower values for more distant cells [6]. Synaptic connections are current-based with an exponential decay and modeled to be in accordance with fast acting glutamatergic and GABAergic neurotransmitters. In order to ease simulation and analysis we restrict our model to have mono-synaptic connections between pairs of cells, aggregating possibly several synaptic contacts of one pair of cells into one postsynaptic potential (PSP).

The E-E (STDP+homeostatic plasticity) and I-E (inhibitory STDP) synaptic connections are dynamic, whereas the I-E and I-I ones are static (see Fig A in S1 Text).

### Synaptic plasticity rules

STDP in our model is implemented in a standard on-line fashion with exponential kernels and all-to-all spike pairings so that the weight update for a synapse connecting a pre- and a postsynaptic cell is given by

$$\begin{array}{c} \Delta w\left(w\right)=\{\begin{array}{cc} A_{+}\left(w\right)\exp\left(-\left(t_{\text{post}}-t_{\text{pre}}\right)/\tau_{+}\right) & \text{if}\;t_{\text{post}}-t_{\text{pre}}>0\\ -A_{-}\left(w\right)\exp\left(\left(t_{\text{post}}-t_{\text{pre}}\right)/\tau_{-}\right) & \text{if}\;t_{\text{post}}-t_{\text{pre}}\leq 0 \end{array}, \end{array}$$

where *t*_pre_ and *t*_post_ denote pre- and postsynaptic spike times.

For excitatory-excitatory synapses, we consider either additive (*A*_+_(*w*) and *A*_−_(*w*) constant), partly multiplicative (*A*_+_(*w*) constant) or fully multiplicative STDP rules, see S1 Text for a description of the different rules. For inhibitory-excitatory synapses we only consider additive rules as in this case additive and multiplicative rules are equivalent [54].

For both excitatory and inhibitory connections, time constants were set to *τ*_+_ = *τ*_−_ = 20 ms. For additive excitatory STDP, the amplitudes of LTP and LTD were chosen as *A*_+_ = 10^−3^ and *A*_−_ = 1.05*A*_+_, respectively, resulting in a negative integral of the STDP window. For the case of partly multiplicative and multiplicative STDP at excitatory synapses we chose *A*_+_ = *A*_−_ = 10^−3^. For inhibitory connections, we set *A*_−_ = 10^−3^ and *A*_+_ = 4*A*_−_ as motivated by experimental findings [44] and yielding a positive integral of the STDP window [54]. In the simulations, *A*_+_ and *A*_−_ are multiplied with the respective maximal weight for additive rules to obtain their effective values.

Like the excitatory STDP rule, inhibitory STDP [44] in our model is Hebbian, increasing the synaptic weight if the postsynaptic cell fires within *τ*_+_ ms after a presynaptic spike, and decreasing it when a presynaptic spike occurs within *τ*_−_ ms after a postsynaptic spike.

We verified that the results do not strongly depend on the learning rates of the STDP rules by varying them one order of magnitude into each direction. This influences the convergence speed of the synaptic weights to the equilibrium distribution, but not the shape of the distribution itself.

Homeostatic plasticity is implemented in form of synaptic weight normalization acting at the postsynaptic site of excitatory-excitatory connections. The normalization rule is defined by

$$\begin{array}{c} w^{\text{scaled}}\left(i\right):=\frac{1}{\sum_{j}w_{j}^{\text{in}}\left(i\right)}\deg_{\text{EE}}^{\text{in}}\left(i\right)w^{\text{in}}\left(i\right), \end{array}$$

where for an excitatory cell *i*, **w** ^in^(*i*) denotes the vector of incoming excitatory weights with components $w_{j}^{\text{in}}(i)$ and ${\text{deg}}_{\text{EE}}^{\text{in}}(i)$ denotes the excitatory in-degree. In the simulations, the above normalization of weights is applied every 100 ms, replacing the weight vector at each cell with its normalized version.

We note that for long simulation times synaptic normalization is equivalent to synaptic scaling, assuming equal mean rates of the presynaptic cells and slow time-scales of plasticity. Yet, we also performed simulations of the network with synaptic scaling mechanisms acting on a slower timescale according to the following rule, analyzed for rate-based models as in [62]:

$$\begin{array}{c} \frac{dw}{dt}=-\gamma \left(\nu -\nu_{0}\right)w^{2}. \end{array}$$

Here, *ν* denotes the firing rate of the cell, *ν*_0_ a target rate and *γ* a learning rate. For the simulations we chose *γ* = 10^−6^ and *ν*_0_ = 0 Hz. A scaling step was performed each 50 ms during the simulation and firing rates were computed using a sliding window of length 100 s. We observed that these networks show qualitatively the same behavior as the ones with the synaptic normalization rule, see Section 12.3 in S1 Text.

### Simulation protocol

At the start of the simulation, the membrane potentials of the neurons were drawn from a uniform distribution between *V*_rest_ and *V*_thres_. Subsequently, the network was driven by a constant depolarization of each cell, sufficient to depolarize each cell by 11 mV. This input drove the network to the asynchronous irregular (AI) regime of activity with a mean population firing rate close to 5 Hz. We chose a constant depolarization as input since we wanted to study self-organization in the network brought about by its own dynamics, rather than some structure present in the input. Additionally, this case was previously studied in [71], where also some properties of the static network were assessed, such as the expression of asynchronous irregular spiking activity.

The distribution of firing rates and interspike intervals (ISIs) in the network is long-tailed with few cells firing at rates up to 30 Hz and many at low rates below 0.1 Hz. As expected, the mean value of the distribution of coefficients of variation of the ISIs is close to 1, indicating irregular spiking activity of the network.

In an alternative setup, we tested a network in which each cell is stimulated by a Poisson spike train of the same mean intensity and noted that this setup results in qualitatively the same results.

We started with a network in which initially all synaptic weights have a constant value of 1 for all four types of synaptic connections between excitatory and inhibitory cells and then activate the synaptic plasticity rules. We also simulated networks with initially Gaussian and uniform weight distributions and obtained qualitatively identical results. After a transient phase lasting around 5 hours of network activity, the weights stabilized to their new long-tailed distributions.

We observed that during this transient phase the network rests in the asynchronous irregular regime of activity. No significant difference in the mean firing rate of the different populations and no apparent visual difference in raster plots before and after plasticity can be observed, see Fig B in S1 Text.

We furthermore verified that the obtained results do not strongly depend on the learning rates chosen by systematically varying them around the chosen value, increasing and decreasing them by up to one order of magnitude. This change in parameters influenced the speed of convergence of the synaptic weights to their equilibrium distributions, but not the shape of the distributions itself, while at the same time taking no influence on network dynamics.

### Analytic results

We studied analytically tractable reduced models that enable us to calculate STDP weight updates in a mean-field fashion and that allow us to give an explanation of the observed processes of self-organization in the network.

#### Steady state inhibitory synaptic weights

Consider a reduced model consisting of two cells that receive Poisson input and that are synaptically connected via an inhibitory delta synapse (i.e. a synapse where each postsynaptic potential is represented by a delta peak) subject to inhibitory STDP. For simplicity of the analysis assume an integrate-and-fire model in the following and that only the nearest neighbor spikes influence STDP.

In this model, assuming small input, the rise of the membrane potential induced by the input is close to linear and the average delay time *d*_*w*_ caused in the postsynaptic spiking by a presynaptic inhibitory spike with connection strength *w* can be calculated as

$$\begin{array}{c} d_{w}\left(w,\nu_{0}\right)=\frac{w}{\nu_{0}\left(V_{\text{thres}}-V_{\text{rest}}\right)}, \end{array}$$ (1)

where *ν*_0_ denotes the firing rate of the postsynaptic cell without the inhibitory connection. Denote by *T*_isi_ = 1/*ν*_0_ the average inter-spike-interval (ISI) of the postsynaptic cell without inhibitory connection. For the following analysis we ignore the variability in the ISIs, using as approximation *T*_isi_. We can now compute the average impact of the nearest preceding and succeeding spike on the synaptic weight *w* as

$$\begin{array}{ccc} \left\langle \Delta w\right\rangle & = & \frac{1}{T_{\text{isi}}}\int_{0}^{T_{\text{isi}}}A_{+}e^{-\frac{T_{\text{isi}}-t+d_{w}}{\tau }}dt-\frac{1}{T_{\text{isi}}}\int_{0}^{T_{\text{isi}}}A_{-}e^{-\frac{t}{\tau }}dt\\ & = & \nu_{0}\tau [A_{+}(e^{-\frac{w}{\nu_{0}\tau \left(V_{\text{thres}}-V_{\text{rest}}\right)}}-e^{-\frac{w+V_{\text{thres}}-V_{\text{rest}}}{\nu_{0}\tau \left(V_{\text{thres}}-V_{\text{rest}}\right)}})+A_{-}(e^{-\frac{1}{\nu_{0}\tau }}-1)]\\ & = & \nu_{0}\tau (1-e^{-\frac{1}{\nu_{0}\tau }})(A_{+}e^{-\frac{w}{\nu_{0}\tau \left(V_{\text{thres}}-V_{\text{rest}}\right)}}-A_{-}), \end{array}$$

where *τ* denotes the time constant of the STDP rule. To find the stationary weight we now solve ⟨Δ*w*(*w*, *ν*_0_)⟩ = 0 for *w* and obtain the stationary weight *w*_stat_(*ν*_0_) as a function of the initial firing rate *ν*_0_. A solution can be obtained in closed form as

$$\begin{array}{c} w_{\text{stat}}\left(\nu_{0}\right)=\nu_{0}\tau \left(V_{\text{thres}}-V_{\text{rest}}\right)\log(\frac{A_{+}}{A_{-}}), \end{array}$$ (2)

see Section 14 in S1 Text for a detailed derivation of Eq 2.

**Fig 9:**
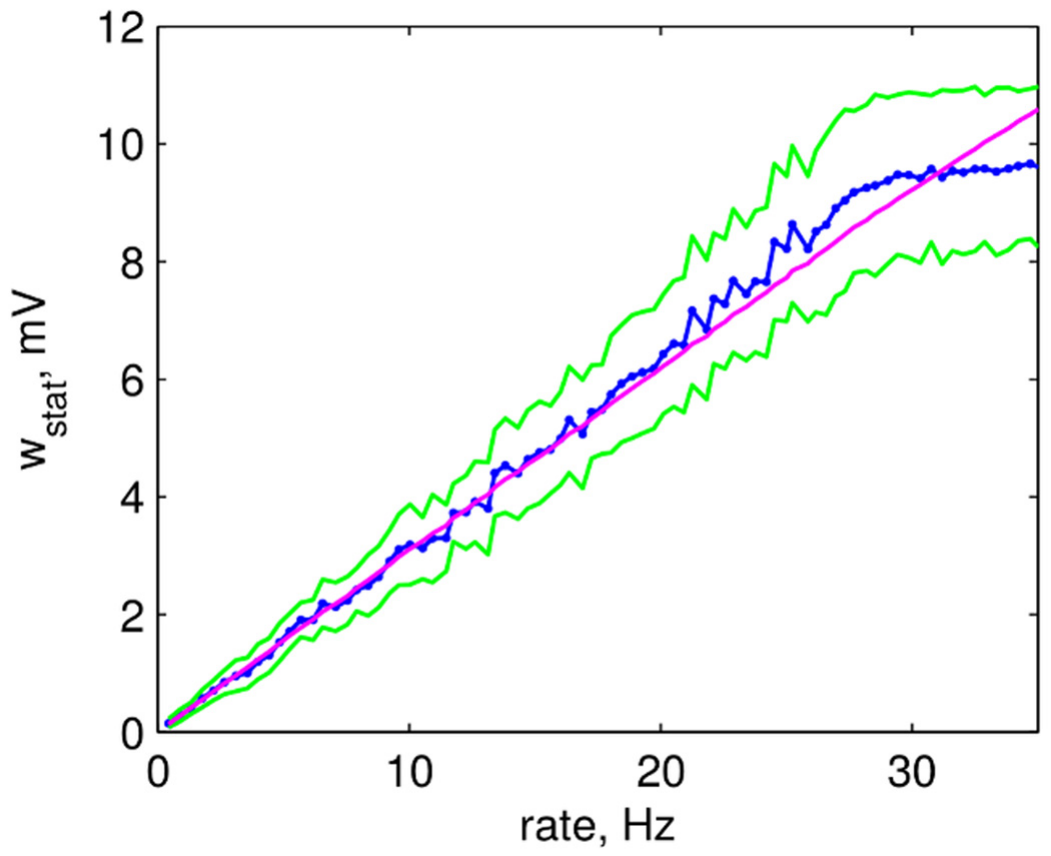
Stationary weight of a plastic inhibitory synapse after plasticity. Average stationary weight after 100 seconds of simulation of a 2-neuron model (blue) and its standard deviation (green) as a function of initial postsynaptic firing rate. Analytic solution for the stationary weight (magenta).

Note that the stationary weight depends only on the quotient of *A*_+_ and *A*_−_, in our case *A*_+_/*A*_−_ = 4. The analytic solution provides a very good fit to the data, Fig 9. Differences observed for large rates are due to the restriction on the maximal inhibitory weight. To find the actual rate *ν* of the postsynaptic neuron, taking into account the inhibitory synapse, we combine Eqs (2) and (1)

$$\begin{array}{c} \nu \left(\nu_{0}\right)=\frac{\nu_{0}}{1+\nu_{\text{inh}}d_{w}\left(w_{\text{stat}}\left(\nu_{0}\right),\nu_{0}\right)}=\frac{\nu_{0}}{1+\tau \nu_{\text{inh}}\log\left(A_{+}/A_{-}\right)}, \end{array}$$

where *ν*_inh_ is the rate of the inhibitory neuron.

If we start driving the postsynaptic neuron stronger, the inhibitory weight will increase, but not as much as needed to compensate the increased drive,

$$\begin{array}{c} 0<\nu \left(\nu_{0}+\Delta \nu_{0}\right)-\nu \left(\nu_{0}\right)=\frac{\Delta \nu_{0}}{1+\tau \nu_{\text{inh}}\log\left(A_{+}/A_{-}\right))}<\Delta \nu_{0}. \end{array}$$

#### Average impact of STDP on a synapse

In our model, excitatory STDP parameters are selected such that depression slightly dominates facilitation. Thus, we expect that very weak synapses will get weaker over time. At the same time, a presynaptic spike of an excitatory neuron shifts the next postsynaptic spike backward in time and thus increases the positive contributions of STDP, allowing the average contribution to increase above 0. Here, we study a mean field model with two cells *A* and *B*, both driven by independent Poisson inputs with the same rate and connected by a delta synapse of strength *w*. We want to find an expression for the average synaptic change Δ as a function of *w*. Given the parameters of the input, it is possible to compute the rates, *ν* = *ν*_*A*_ = *ν*_*B*_ and membrane potential distributions *P*(*V*) of both cells [35], see Section 6 in S1 Text.

**Fig 10:**
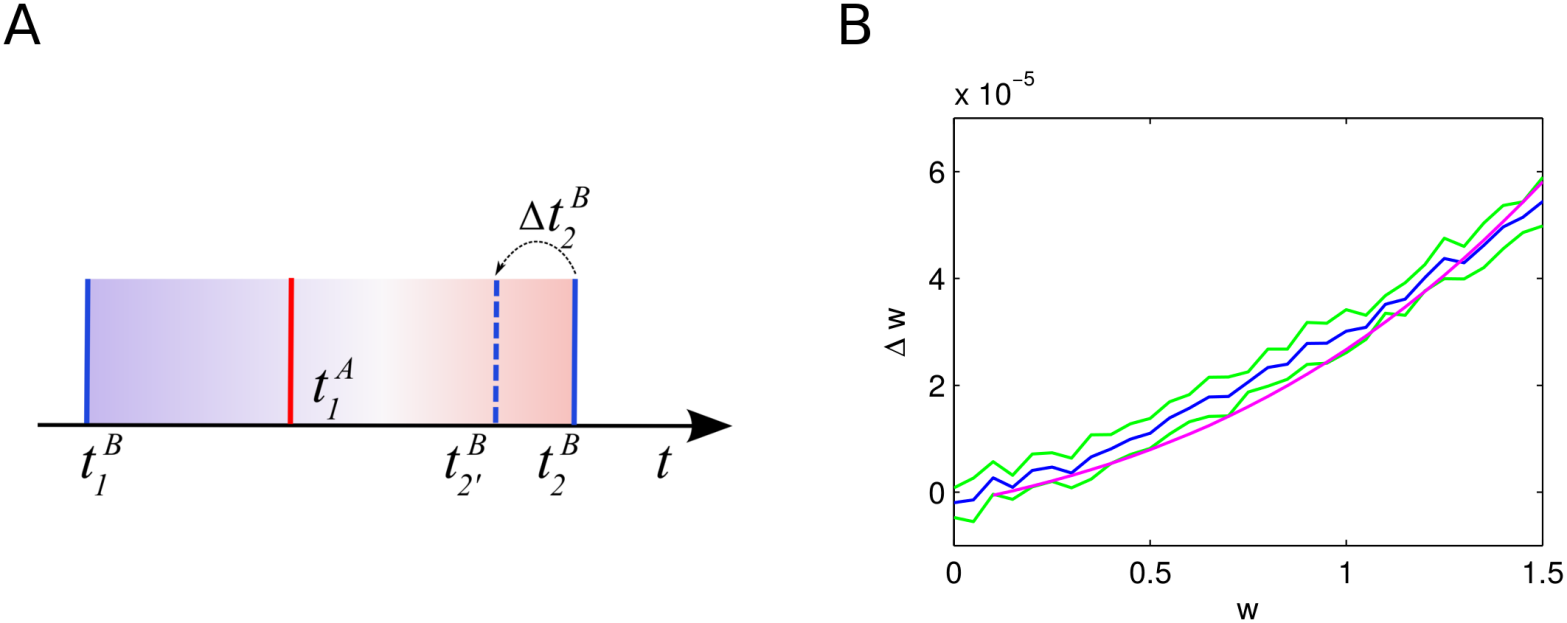
Average change in synaptic weight due to a STDP update depending on the initial synaptic strength. **A**: Sketch of the three-spikes scenario, spikes of the postsynaptic neuron are shown in blue, spikes of the presynaptic neuron in red. **B**: Average change in synaptic strength Δ*w* depending on the initial synaptic strength *w* (blue curve), standard deviation based on 10 simulations of 500 seconds (green curve) and solution of Eq 6 (magenta curve).

For simplicity, we again only consider nearest spikes STDP interactions here. To approximate Δ*w*, it is enough to consider triplets of spikes: one presynaptic spike between two postsynaptic ones. Let neuron *B* fire at times $t_{1}^{B}$ and $t_{2}^{B}$ and neuron *A* fire at time $t_{1}^{A}$ such that $t_{1}^{B}<t_{1}^{A}<t_{2}^{B}$, see Fig 10. We denote by STDP^+^(Δ*t*) = *A*_+_exp(−Δ*t*/*τ*_+_) and STDP^−^(Δ*t*) = −*A*_−_exp(Δ*t*/*τ*_−_) the positive and negative parts of STDP-kernel, respectively, where Δ*t* denotes the time-difference between spikes of the postsynaptic and the presynaptic neuron. Consider in particular the inter-spike interval of cell *B* given by $T=t_{2}^{B}-t_{1}^{B}$. Ignoring the impact of a presynaptic spike on the membrane potential, we can compute:

$$\begin{array}{c} \Delta w\left(T\right)=\int_{0}^{T}{\text{STDP}}^{+}\left(T-t\right)-{\text{STDP}}^{-}\left(-t\right)dt. \end{array}$$ (3)

Additionally, the presynaptic spike leads to a time-shift in the firing of the postsynaptic neuron. To compute the average time-shift $\Delta t_{2}^{B}$ we use the change in the rate Δ*r* ^*B*^(*w*) of the postsynaptic neuron expressed as a function of the synaptic weight:

$$\begin{array}{c} \Delta t_{2}^{B}\left(w\right)=\frac{\Delta r^{B}\left(w\right)}{r^{B}(r^{B}+\Delta r^{B}\left(w\right))}. \end{array}$$

Combining this equation with Eq 3, we can obtain a better approximation of Δ*w*(*T*)

$$\begin{array}{c} \Delta w\left(T\right)=\int_{\Delta t_{2}^{B}}^{T-\Delta t_{2}^{B}}{\text{STDP}}^{+}\left(T-t-\Delta t_{2}^{B}\right)-{\text{STDP}}^{-}\left(-t\right)dt+\int_{0}^{\Delta t_{2}^{B}}{\text{STDP}}^{+}\left(T-t\right)-{\text{STDP}}^{-}\left(-t\right)dt, \end{array}$$ (4)

where here we did not consider the interval $t\in (T-\Delta t_{2}^{B},T]$, that corresponds to cases where a presynaptic spike could trigger the postsynaptic neuron to fire. Now we need to take into account a distribution of inter-spikes-intervals *T*. For a perfect Poisson spike train the inter-spike intervals would be exponentially distributed, but for Poisson-driven leaky integrate-and-fire neurons the inter-spike-interval distribution is better approximated by an inverse Gaussian distribution [89] *T* ∼ *IG*(*μ*, *λ*), where *μ* = 1/*ν*.

Finally, a presynaptic spike can directly trigger a postsynaptic spike. The probability of such an event is easy to compute from the distribution of membrane potentials:

$$\begin{array}{c} P^{\text{fire}}\left(w\right)=\int_{V^{\text{thr}}-w}^{V^{\text{thr}}}P\left(V\right)dV. \end{array}$$ (5)

see Section 6 in S1 Text.

Combining the previous computations, we obtain the estimation of Δ*w* as:

$$\begin{array}{c} \Delta w=\int_{0}^{\infty }P\left(T\right)(\int_{\Delta t_{2}^{B}}^{T-\Delta t_{2}^{B}}{\text{STDP}}^{+}\left(T-t-\Delta t_{2}^{B}\right)-{\text{STDP}}^{-}\left(-t\right)dt+\\ \int_{0}^{\Delta t_{2}^{B}}{\text{STDP}}^{+}\left(T-t\right)-{\text{STDP}}^{-}\left(-t\right)dt)dT+P^{\text{fire}}\left(w\right){\text{STDP}}^{+}\left(\tau_{\text{syn}}\right), \end{array}$$ (6)

where *τ*_syn_ denotes the synaptic time delay.

To check the accuracy of Eq 6, we simulated a two-neuron model. Both neurons are driven by Poisson input from 1650 delta synapses with a rate of 1 Hz and connection strengths of 0.5 mV. We found that Δ*r* ^*B*^(*w*) varies linearly with *w*, and use the parameters of the linear fit from the data to obtain Δ*r* ^*B*^(*w*). The parameter *λ* of the inverse Gaussian distribution for inter-spikes intervals we found not to influence the results of the estimation of Δ*w*, at least in the interval 0.5/*ν* < *λ* < 1/*ν*. Using Eq 6, we obtain a good fit to the simulated data, see Fig 10.

## Supporting Information

S1 Text Appendix containing further details and derivation of analytical results. (PDF)
