# Supplementary material for: Self-organization in Balanced State Networks by STDP and Homeostatic Plasticity
Source: PLoS Comput Biol. 2015 Sep 3;11(9):e1004420. doi: 10.1371/journal.pcbi.1004420 (PMC4559467; doi:10.1371/journal.pcbi.1004420)
Supplement: S1 Text — (PDF) [file pcbi.1004420.s001.pdf]

Self-organization in balanced state networks by STDP and homeostatic plasticity – Appendix

Felix Effenberger, Jürgen Jost, Anna Levina

Contents

1 Model description **2**

2 Network topology, weight distributions and dynamics **3**

3 STDP in the model **4**

4 Interplay of excitatory STDP and synaptic scaling **5**

5 Inhibitory currents on driver cells **6**

6 Analytical approximation using a Fokker-Planck formalism **6**

7 Network without inhibitory STDP **7**

8 Homogeneous networks and under-inhibition **9**

9 Varying the driver threshold **10**

10 Varying network size **10**

11 Topographic networks **11**

12 Different learning rules **11**
12.1 Partly multiplicative STDP 12
12.2 Multiplicative STDP 13
12.3 Synaptic scaling 15

13 Clustering of strong synapses **16**

14 Derivation of stationary inhibitory weights **17**

1 Model description

A. Model Summary	
Populations	Two: excitatory (E), inhibitory (I)
Topology	—
Connectivity	Random divergent connections
Neuron model	Leaky integrate and fire (LIF), fixed voltage threshold, fixed absolute refractory time (voltage clamp)
Channel models	—
Synapse model	exponential synapses
Plasticity	additive STDP for E-E and I-E synapses
Input	constant depolarization to -49 mV or independent fixed-rate Poisson spike trains to all neurons
Measurement	Spiking activity, excitatory and inhibitory currents

B. Populations		
**Name**	**Elements**	**Size**
E	LIF neuron	$N_\mathrm{E} = 4N_\mathrm{I}$
I	LIF neuron	N_I

C. Connectivity			
**Name**	**Source**	**Target**	**Pattern**
EE	E	E	Random divergent $1 \to C_\mathrm{E}$, weight w_EE, delay $d = 1.5\,\mathrm{ms}$, $C_\mathrm{E} = 0.02 \cdot N_\mathrm{E}$
EI	E	I	Random divergent $1 \to C_\mathrm{I}$, weight w_EI, delay d, $C_\mathrm{I} = 0.02 \cdot N_\mathrm{I}$
IE	I	E	Random divergent $1 \to C_\mathrm{E}$, weight w_IE, delay d
II	I	I	Random divergent $1 \to C_\mathrm{I}$, weight w_II, delay d

D. Neuron model	
Name	LIF neuron
Type	Leaky integrate and fire, current-based synapses
Subthreshold dynamics	$\tau_\mathrm{m}\frac{dV}{dt} = -(V - E_\mathrm{L}) + I_\mathrm{E}^\mathrm{syn} - I_\mathrm{I}^\mathrm{syn}, I_\mathrm{E}^\mathrm{syn} = w_\mathrm{E} c_\mathrm{E}^\mathrm{norm} g_\mathrm{E}$, with $I_\mathrm{E}^\mathrm{syn} = w_\mathrm{E} c_\mathrm{E}^\mathrm{norm} g_\mathrm{E}$, $c_\mathrm{E}^\mathrm{norm} = 1\,\mathrm{mV}$, $I_\mathrm{I}^\mathrm{syn} = w_\mathrm{I} c_\mathrm{I}^\mathrm{norm} g_\mathrm{I}$, $c_\mathrm{I}^\mathrm{norm} = -9\,\mathrm{mV}$, $\tau_\mathrm{m} = 20\,\mathrm{ms}$, $E_\mathrm{L} = -60\,\mathrm{mV}$.
Spiking	If $V(t-) < V_\mathrm{thres}$ and $V(t+) \geq V_\mathrm{thres} = -50\,\mathrm{mV}$, set $t^* = t$, emit spike with time stamp t^* and clamp $V(t) = V_\mathrm{reset} = -60\,\mathrm{mV}$ for $\tau_\mathrm{ref} = 2\,\mathrm{ms}$.

G. Synapse models	
excitatory	$\tau_\mathrm{E}\frac{dg_\mathrm{E}}{dt} = -g_\mathrm{E}$, $\tau_\mathrm{E} = 5\,\mathrm{ms}$
inhibitory	$\tau_\mathrm{I}\frac{dg_\mathrm{I}}{dt} = -g_\mathrm{I}$, $\tau_\mathrm{I} = 10\,\mathrm{ms}$

F. Plasticity models	
STDP	$\Delta w = A_+ \exp(-(t_{\text{post}} - t_{\text{pre}})/\tau_+)$ if $t_{\text{post}} - t_{\text{pre}} > 0$ else $\Delta w = -A_- \exp((t_{\text{post}} - t_{\text{pre}})/\tau_-)$.
STDP at EE	$A_+ = 10^{-3}$, $A_- = 1.05A_+$, $w_{\text{E}}^{\max} = 20$, initial synaptic weight $w_{\text{E}} = 1$ or drawn from uniform distribution on $[0, w_{\text{E}}^{\max}]$, $\tau_+ = \tau_- = 20\,\text{ms}$
STDP at IE	$A_- = 2 \cdot 10^{-3}$, $A_+ = 4A_-$, $w_{\text{I}}^{\max} = 5$, initial synaptic weight $w_{\text{I}} = 1$ or drawn from uniform distribution on $[0, w_{\text{I}}^{\max}]$, $\tau_+ = \tau_- = 20\,\text{ms}$
Scaling at EE	$\mathbf{w}^{\text{scaled}}(i) := \frac{1}{\sum_j w_j^{\text{in}}(i)} \deg_{\text{EE}}^{\text{in}}(i)\mathbf{w}^{\text{in}}(i)$, where $\mathbf{w}^{\text{in}}(i)$ denotes the vector of incoming excitatory weights with components $w_j^{\text{in}}(i)$ at excitatory cell i and $\deg_{\text{EE}}^{\text{in}}(i)$ its excitatory in-degree.

2 Network topology, weight distributions and dynamics

We consider a random, balanced state network of excitatory and inhibitory leaky integrate and fire neurons. In our model, E-E (STDP+homeostatic plasticity) and I-E (inhibitory STDP) synaptic connections are dynamic, whereas the I-E and I-I ones are static (see Fig. A).

Synaptic in- and out-degrees of cells are binomially distributed with mean degrees of synaptic connectivity for the different cell types given by $\overline{\deg}_{\text{EE}}^{\text{out}} = \overline{\deg}_{\text{EE}}^{\text{in}} = \overline{\deg}_{\text{IE}}^{\text{out}} = 0.02 \cdot 4000 = 80$ and $\overline{\deg}_{\text{II}}^{\text{out}} = \overline{\deg}_{\text{II}}^{\text{in}} = \overline{\deg}_{\text{IE}}^{\text{in}} = 0.02 \cdot 1000 = 20$, where the subscript XY in $\overline{\deg}_{\text{XY}}$ denotes the type of the connection from population X to population Y and the superscript denotes the degree type, $\overline{\deg}^{\text{in}}$ and $\overline{\deg}^{\text{out}}$ denoting the average in-degree and out-degree, respectively.

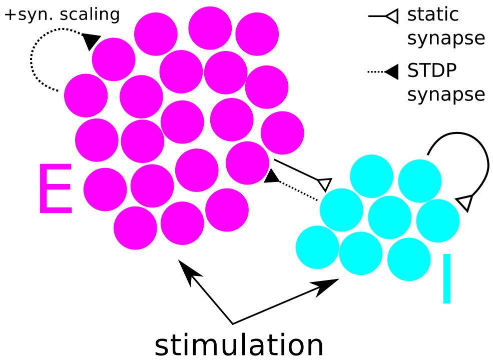

Figure A: Schema of network architecture with static and dynamic synaptic connections. The excitatory population is marked with E, the inhibitory population with I.

Each cell receives input resulting in a constant depolarization by 11 mV. This drives the network to the asynchronous irregular (AI) state, cf. [1]. The network displays long-tailed firing rate distributions with a mean firing rate of around 5 Hz (see Fig. B, left column).

After switching on the plasticity rules, the network enters a transient phase in which excitatory synaptic weights converge to a long-tailed distribution. During the transient phase the network rests in the AI regime of activity. After the weights have settled to their new equi-

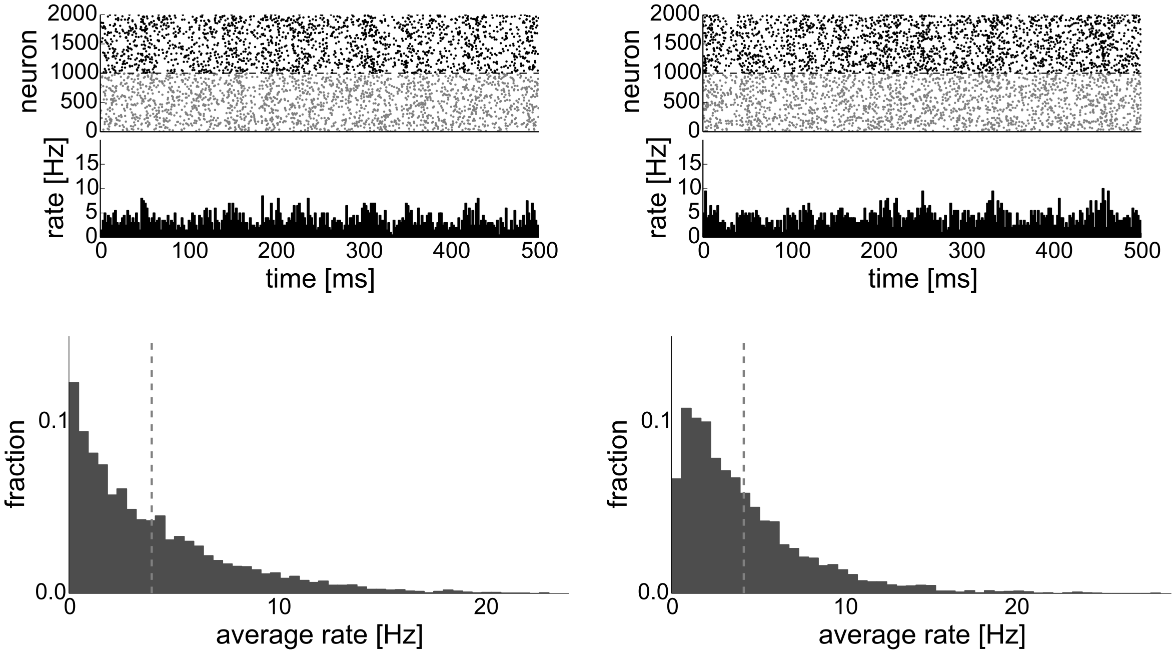

Figure B: Network activity before plasticity (left column) and after plasticity (right column). Top row: Raster plots of 1000 randomly sampled excitatory (black) and inhibitory (gray) cells. Bottom row: Firing rate distributions.

librium, the network still expresses long-trailed distributions of firing rates and rests in the AI regime (see Fig. B, right column).

3 STDP in the model

STDP in our model is implemented in an on-line fashion as a pair-wise rule with all-to-all pairing. Its implementation is as follows.

Denote by x and y the traces of spikes of a given pair of a pre- and a postsynaptic cell, respectively. Whenever a pre- or postsynaptic spike occurs, x and y are increased by quantities $a_+(x)$ and $a_-(y)$ and decay as specified by time constants τ_+ and τ_-:

$$\tau_+ \frac{dx}{dt} = -x + a_+(x) \sum_i \delta(t - t_i) \quad \text{and} \quad \tau_- \frac{dy}{dt} = -y + a_-(y) \sum_j \delta(t - t_j).$$

Here, δ is the δ-function and t_i and t_j denote times of pre- and postsynaptic spikes.

An STDP rule altering the synaptic weight w of a synapse connecting the pre- and the postsynaptic cell can then be written as

$$\frac{dw}{dt} = A_+(w) x(t) \sum_j \delta(t - t_j) - A_-(w) y(t) \sum_i \delta(t - t_i),$$

where $A_+(w)$ and $A_-(w)$ are functions describing the magnitudes of synaptic depression and facilitation, respectively.

STDP rules for which the magnitude of $A_+(w)$ and $A_-(w)$ are independent of the weight w are called *additive* and can be written as

$$A_+(w) = \Theta(w_{\max} - w)\eta_+ \quad \text{and} \quad A_-(w) = \Theta(w - w_{\min})\eta_-,$$

where $w_{\min} = 0$ and $w_{\max}$ denote chosen minimal and maximal synaptic weights and where Θ is the Heaviside step function.

STDP rules for which either $A_+(w)$ or $A_-(w)$ multiplicatively depend on w are called *partly multiplicative*. One particular form characterized by an additive potentiation and a multiplicative depression is often called *van Rossum STDP* [2]. It can be written as

$$A_+(w) = \Theta(w_{\max} - w)\eta_+ \quad \text{and} \quad A_-(w) = w\eta_-.$$

Unless stated otherwise, we use additive STDP rules with $a_+(x) = a_-(y) = 1$ and $\tau_+ = \tau_- = 20$ ms throughout the simulations. Furthermore, we use $\eta_+ = 10^{-3}$, $\eta_- = 1.05 \cdot 10^{-3}$, $w_{\min} = 0$, $w_{\max} = 20$ for excitatory STDP and $\eta_+ = 4 \cdot 10^{-3}$, $\eta_- = 10^{-3}$, $w_{\min} = 0$, $w_{\max} = 5$ for inhibitory STDP. Learning rates η_+, η_- are multiplied with the maximal weight for additive rules to obtain their effective values.

4 Interplay of excitatory STDP and synaptic scaling

In order to understand the dynamics of the excitatory STDP rule and its interplay with the included homeostatic plasticity that leads to the emergence of driver neurons in our base model, we consider a reduced model in which 80 presynaptic neurons are connected to one postsynaptic neuron (this is the mean number of incoming excitatory connections in our base model). The synapses are as in the base model and subject to additive STDP (see Section 3). The postsynaptic neuron implements a synaptic normalization as described in the main text.

We observe that in a setting where all cells receive Poisson input of the same intensity such that they spike irregularly at rates of around 5 Hz, a random subset of the synaptic weights converges to the maximum while most synaptic weights remain small (data not shown).

In a setting where a number of presynaptic cells receive stronger input and fire at higher rates of around 25 Hz (close to the rates of driver cells in the base model) we observe that these cells develop strong outgoing synapses converging to the maximal synaptic weight in almost all cases (see Fig. 4). The faster firing cells win the competition over the limited total amount of synaptic weight available on the postsynaptic site (due to the weight normalization). This in turn is due to a fact that strong enough synapses are on average growing with each presynaptic spike, and thus neurons that fire more will get their synapses more potentiated in the same interval of time. For analytical results on this phenomenon see Methods in main text.

5 Inhibitory currents on driver cells

Driver cells in our model receive lower inhibitory currents (see Fig. C). This is a result of a lower inhibitory in-degree of those cells as well as reduced firing rates of the presynaptic inhibitory cells when compared to the network baseline (see Fig. D).

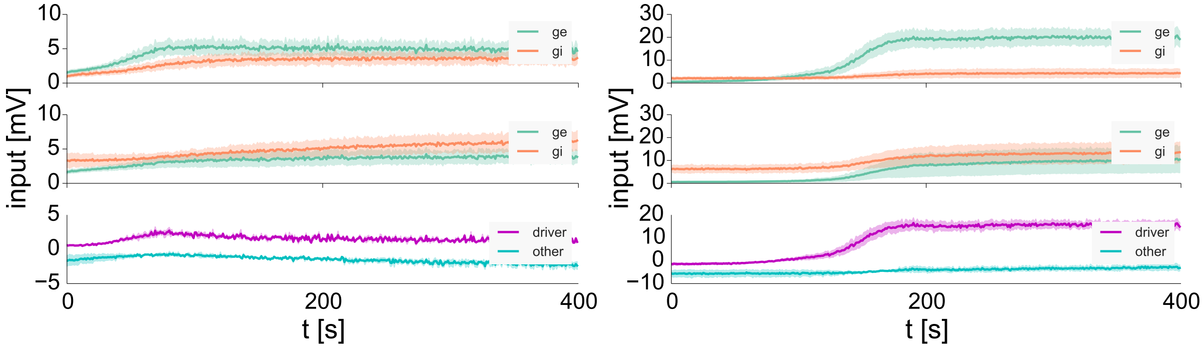

Figure C: Evolution of excitatory (ge) and inhibitory inputs (gi) to driver cells and randomly sampled cells. Left: network with inhibitory STDP, right: network without inhibitory STDP. In each plot from top to bottom: input to driver cells, input to randomly sampled cells, difference of excitatory and inhibitory inputs to driver and random cells. Solid lines represent mean over 10 trials, shaded areas the standard deviation.

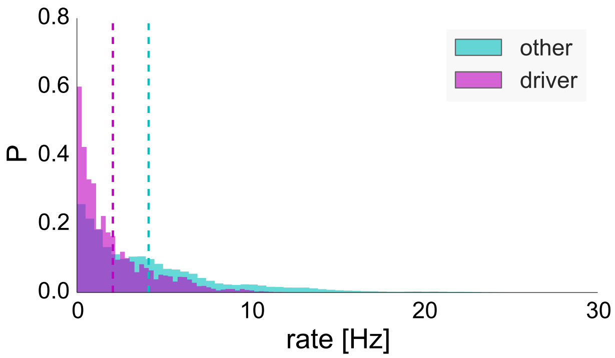

Figure D: Firing rates of inhibitory neurons presynaptic to driver cells and presynaptic to random cells. Dashed lines mark means.

6 Analytical approximation using a Fokker-Planck formalism

In this section we mainly follow the lines of Nicolas Brunel's computations [3, 4]. The steady state membrane potential distribution of a leaky integrate-and-fire (LIF) neuron driven by Poisson input modeled as delta pulses is given by [4]

$$P(V) = \frac{2\nu\tau_{\rm m}}{\sigma} \exp\left(-\frac{(V-\mu)^2}{\sigma^2}\right) \int_{\frac{V-\mu}{\sigma}}^{\frac{V_{\rm th}-\mu}{\sigma}} \Theta\left(u - \frac{V_{\rm r}-\mu}{\sigma}\right) \exp(u^2) du, \tag{1}$$

where Θ denotes the Heaviside step function, $\tau_{\rm m}$ denotes the membrane time constant, $V_{\rm th}$ the threshold potential, $V_{\rm r}$ the reset potential and $\mu = C_e J \tau \nu_{\rm ext}$ and $\sigma = \sqrt{\mu J}$ the mean and variance of the external input (C_e denotes the number of external connections, J the weight of the external connections and $\nu_{\rm ext}$ the mean external firing rate) to each neuron and ν its mean firing frequency defined via the consistency equation

$$\int_{-\infty}^{V_{\rm th}} P(V)dV + \nu\tau_{\rm ref} = 1, \tag{2}$$

that yields for the mean firing rate

$$\frac{1}{\nu} = \tau_{\rm ref} + \tau_{\rm m}\sqrt{\pi} \int_{\frac{V-\mu}{\sigma}}^{\frac{V_{\rm th}-\mu}{\sigma}} \exp(u^2)(1 + \mathrm{erf}(u))du, \tag{3}$$

with $\tau_{\rm ref}$ denoting the absolute refractory period of the model neuron and $\mathrm{erf}(u) = 2/\sqrt{\pi}\int_0^u \exp x^{-2} dx$ denoting the error function. See Figure E(left) for an example of such a distribution of membrane potentials.

In this setting, let us assume that two cells that are synaptically coupled via an excitatory delta synapse with amplitude w. The probability of a presynaptic spike to emit a postsynaptic one is then given by

$$P_{\rm spike}(w) = \int_{V_{\rm th}-w}^{V_{\rm th}} P(v)dv, \tag{4}$$

assuming stationarity in the firing rates of both cells and that the effect of the presynaptic spikes does not take strong influence on the firing rate and membrane potential distribution $P(V)$ of the postsynaptic cell. See Figure E(right) for an example of the distribution of $P_{\rm spike}(w)$ assuming a distribution of V according to Figure E(left) with respect to a synaptic weight w of the connecting synapse.

Does Eq. 4 suffice to explain the emergence of driver neurons? The answer is no and this is due to the negativity of the integral of the STDP curve: Although each presynaptic spike at a synapse with weight $w > 0$ causes the postsynaptic neuron to cross threshold and spike with non-zero probability, this can happen at any instant and taking the integral over time still yields an LTD-dominated change in synaptic weight. An analytical model that seeks to explain the emergence of driver neurons has to consider the shift in spike times of postsynaptic neurons caused by presynaptic spikes. We developed such a model in the main text, see Methods.

7 Network without inhibitory STDP

Network dynamics in our model are highly sensitive to over-excitation due to increases in excitatory synaptic weights. Without inhibitory STDP, the population firing rate of the excitatory

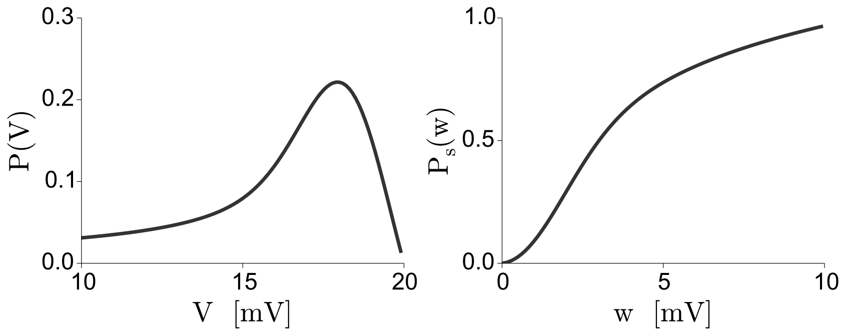

Figure E: Membrane potetial distribution obtained from a mean-field approximation. Left: Probability distribution $P(V)$ of the membrane potential V of a LIF cell driven by Poisson input. $V_{\mathrm{r}} = 10\,\mathrm{mV}$, $V_{\mathrm{th}} = 20\,\mathrm{mV}$, $J = 0.2$,mV, $\nu_{\mathrm{ext}} = 1.2\,\mathrm{Hz}$ resulting in a firing rate of $\nu = 14$ Hz. Right: Probability $P_S(w)$ of a presynaptic spike causing a postsynaptic one assuming a synaptic weight of w and the membrane potential distribution shown on the left.

population quickly decouples from the inhibitory one and the whole excitatory population fires at elevated rates (see Fig. F). In particular, excitatory cells postsynaptic to *future driver cells* (i.e. cells that receive less than average inhibitory currents and thus fire at higher than average rates) receive a higher excitatory drive due to the increasing excitatory weights converging onto those cells and the high firing rates of driver cells. This results in growing excitatory weights of those cells. As a consequence, their synapses compete with synapses of driver cells for the available postsynaptic weight. As a result, only a fraction of the synapses diverging from possible future driver cells can converge to their maximal values, resulting in the cells not becoming driver cells. Without inhibitory STDP, excitatory weight distributions are still long-tailed but strong excitatory weights in the network are more homogeneously distributed rather than being clustered on driver cells. This situation is characterized by many excitatory cells having some strong outgoing synapses (see Fig. G).

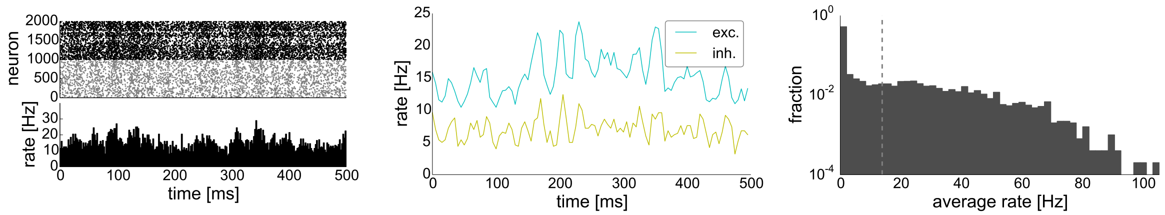

Figure F: Network dynamics without inhibitory plasticity. Left: Raster plot of 1000 randomly sampled excitatory (black) and inhibitory (grey) cells. Middle: Population firing rates of excitatory and inhibitory population. Right: Distribution of firing rates.

In the base model the results stay qualitatively the same if the learning rates of the inhibitory STDP rule are altered. We tested values between $\eta_+ = 3\eta_-$ and $\eta_+ = 5\eta_-$. We observe that with a decreasing quotient of η_-/η_+ the spread of the excitatory weight distribution also decreases.

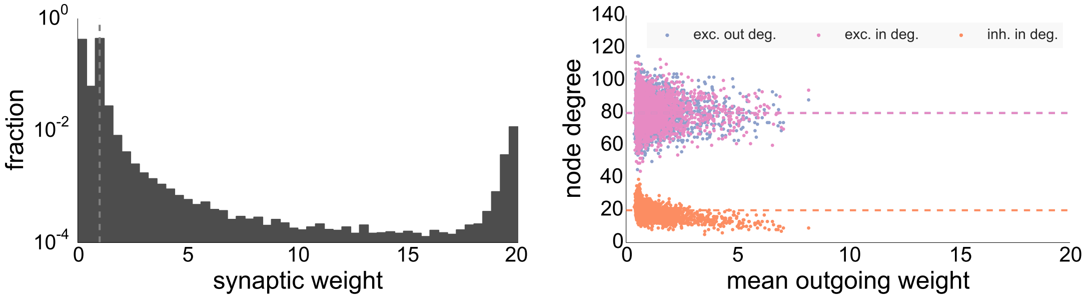

Figure G: Weight distributions in model without inhibitory plasticity. Left: Excitatory weight distribution. Right: Mean values of outgoing excitatory weights per excitatory cell versus node degrees. Dashed lines indicate means.

Fig. H shows the synchrony triggered averages for coincident driver and non-driver spiking in the network. Although joint spiking events in the cells with the strongest outgoing means have a detectable effect on network dynamics, it is much less pronounced than in the configuration including inhibitory STDP.

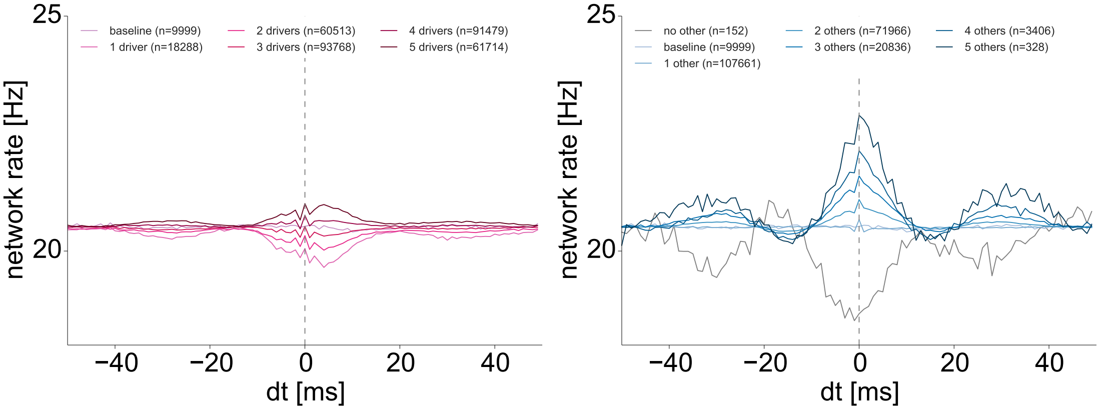

Figure H: Synchrony triggered averages for driver neurons (left) and group of 20 randomly selected non-driver neurons (right) in network without inhibitory STDP. Traces are averages of the excitatory population firing rate and a vertical dashed line marks the 2 ms time bin of synchronous spiking.

8 Homogeneous networks and under-inhibition

In order to show that inhomogeneities in network connectivity are causal to the emergence of driver cells, we simulated a fully homogeneous network in which all cells have the same in-degrees. In this case, the weight distributions are almost delta peaks, i.e. each synaptic weight remains $w \approx 1$ even subject to plasticity and no driver cells emerge, see Fig. I(left).

Interestingly, already a slight amount of under-inhibition suffices to allow for the emergence of driver neurons. We demonstrate this by selecting a group of 50 cells in the fully homogeneous network and selectively pruning 10% of the inhibitory synapses converging onto each cell of the group. This small change in homogeneity suffices to allow the group to become driver cells, see Fig. I(right).

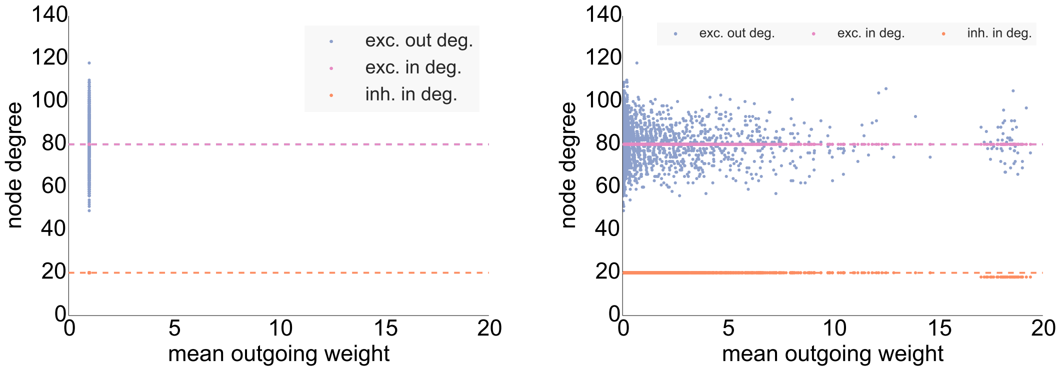

Figure I: Mean outgoing weights versus node degrees of excitatory cells. Left: Network with homogeneous connectivity, all cells have the same in-degree. Right: Network with partially homogeneous connectivity, all cells but a subgroup of 50 have the same in-degree. Each cell of the subgroup has 10% less inhibitory synapses converging than cells of the rest of the network.

9 Varying the driver threshold

As discussed in the main text, we can vary the threshold for being a driver cell a bit and still get qualitatively similar results regarding the dynamical impact of driver cell spiking on network activity. To assess this, instead of selecting as driver cells the top 0.5% (amounting to $n = 20$) of the cells with the strongest outgoing mean weights, we selected the top 3% (amounting to $n = 120$) and computed the synchrony triggered averages for this bigger group as described in the main text. We find qualitatively the same results, see Fig. J.

10 Varying network size

In order to test sensitivity of the findings with respect to network size, apart from the network size of $5,000$ neurons that we used in the manuscript, we additionally simulated networks with $10,000$ (see Fig. K) and $20,000$ (see Fig. L) neurons. We find in both cases that the emergent network properties described in the main text (long-tailed weight distributions, driver neurons) still hold, albeit absolute mean outgoing weights of the driver cell group decrease a bit with network size. This effect can be explained with the tendency towards lower firing rates in bigger

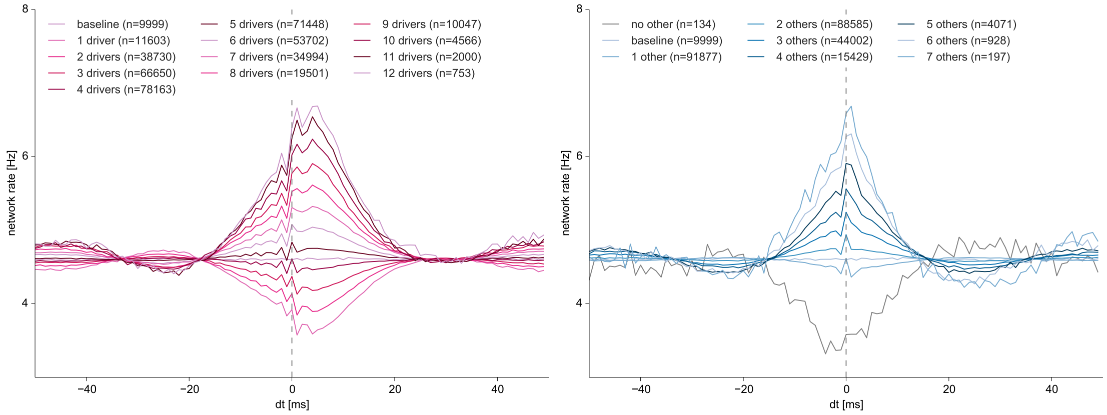

Figure J: Synchrony triggered averages for driver neurons (left, where the top 3%, i.e. 120 neurons, of all excitatory cells with the highest outgoing mean weight are selected to be drivers) and group of 120 randomly selected non-driver neurons. Traces are averages of the excitatory population firing rate and a vertical dashed line marks the 2 ms time bin of synchronous spiking.

networks (see top right panels in Figs. K, L) which is causal for driver emergence as we show in the main text.

11 Topographic networks

Networks with local, distant-depended connectivity profiles are often taken as models for cortical connectivity [5]. To verify whether our findings hold for such types of networks, we simulated networks consisting of $10,000$ cells on the torus as described in the main text.

Here, we show some of their properties for the case of additive STDP rules. The results for partly or fully multiplicative STDP rules at excitatory synapses qualitatively look like the ones of the corresponding random networks.

The weights and rates distributions are very similar to the ones observed in the random network: rates are distributed approximately log-normal, the largest part of the weights distribution follows a power-low, see Fig. M.

12 Different learning rules

In order to assess the generality of the model, we exchange the plasticity rules and see if we obtain results (dynamical state, weight distributions, clustering of strong weights and emergence of driver neurons) qualitatively similar to the base model.

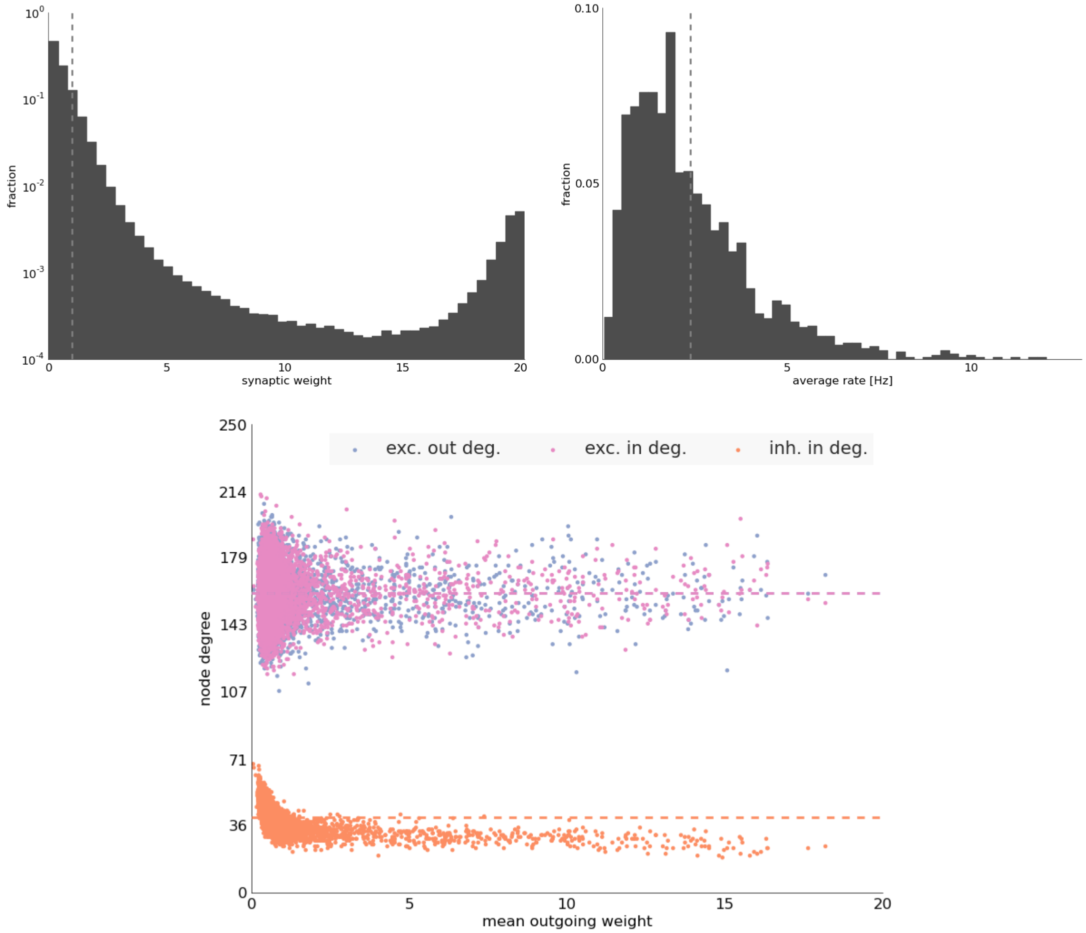

Figure K: Network with 10, 000 neurons. Top: Distribution of excitatory synaptic weights and firing rates. Bottom: Mean outgoing weight of excitatory cells versus connection degrees.

12.1 Partly multiplicative STDP

Exchanging the additive STDP rule at E-E synapses with a partly multiplicative one characterized by additive potentiation and multiplicative depression [2] ("van Rossum STDP", see Section 3) results in a network that still expresses long tailed distributions of firing rates and long-tailed distributions of the excitatory weights, albeit this distribution being much less widespread than in the base model, Fig. N. As in the previous studies [2], we observe that synaptic weights distribution becomes unimodal, and the tail is no longer power-law distributed, but log-normal. Here, the largest EPSP sizes are much smaller than the largest ones in base model. This is due to the fact that in contrast to additive STDP, stronger synapses are subject to much stronger LTD under this rule [2].

Yet we still observe a clustering of the strongest outgoing synapses on cells with higher firing rates (see Fig. O, right), leading to the emergence of driver cells with a high mean outgoing

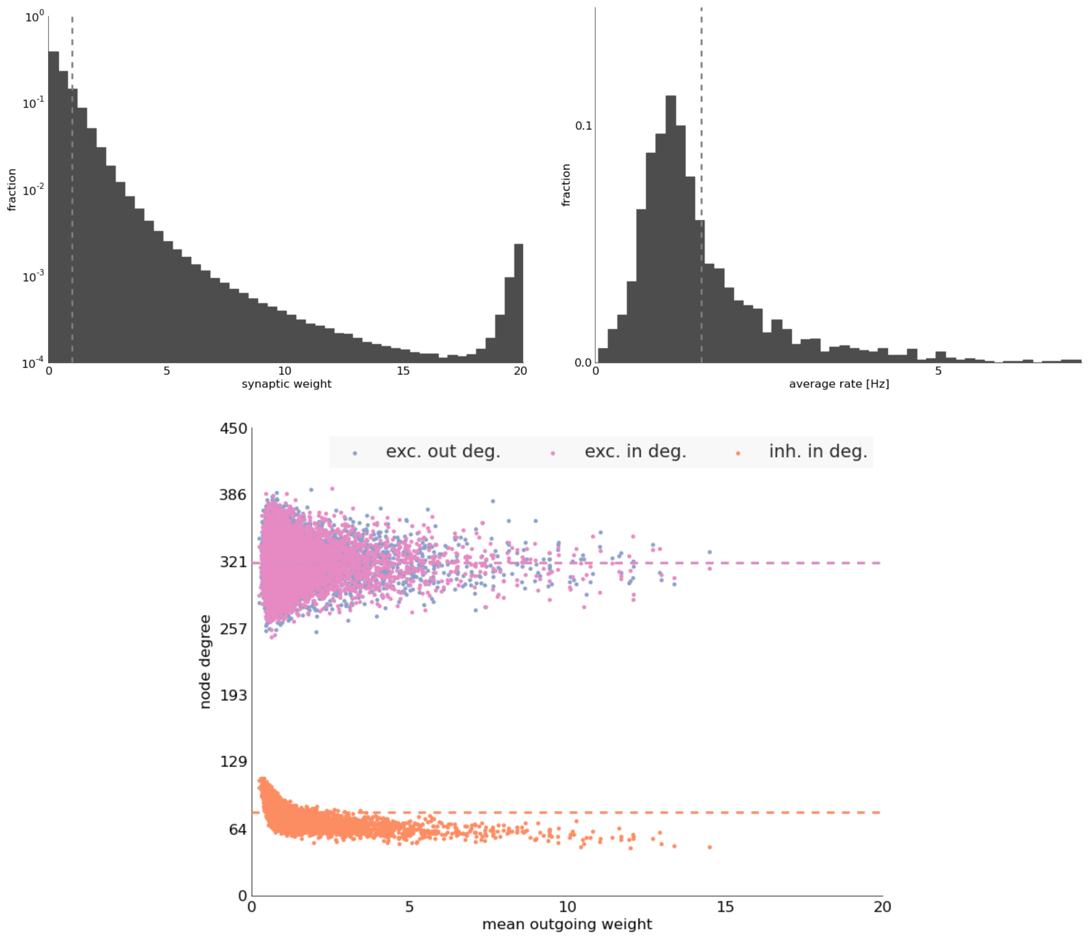

Figure L: Network with 10, 000 neurons. Top: Distribution of excitatory synaptic weights and firing rates. Bottom: Mean outgoing weight of excitatory cells versus connection degrees.

weight. So although in this case the dynamical effect of driver neurons on their postsynaptic networks is much less pronounced due to the smaller absolute synaptic weights compared to the base model, they still exist (see Fig. P).

For the simulations we set $\eta_+ = \eta_- = 10^{-3}$.

12.2 Multiplicative STDP

Exchanging the additive STDP rule at E-E synapses with a fully multiplicative one (see Section 3) results in a network expressing similar rate and weight distributions as in the case of a van Rossum STDP rule described previously, see Section 12.1.

The weight distribution again is unimodal with a log-normal tail, see Fig. Q. Here again, (a) the largest EPSP sizes are much smaller than the largest ones in the model with additive STDP due to stronger effects of LTD on strong synapses and (b) we observe a clustering of the

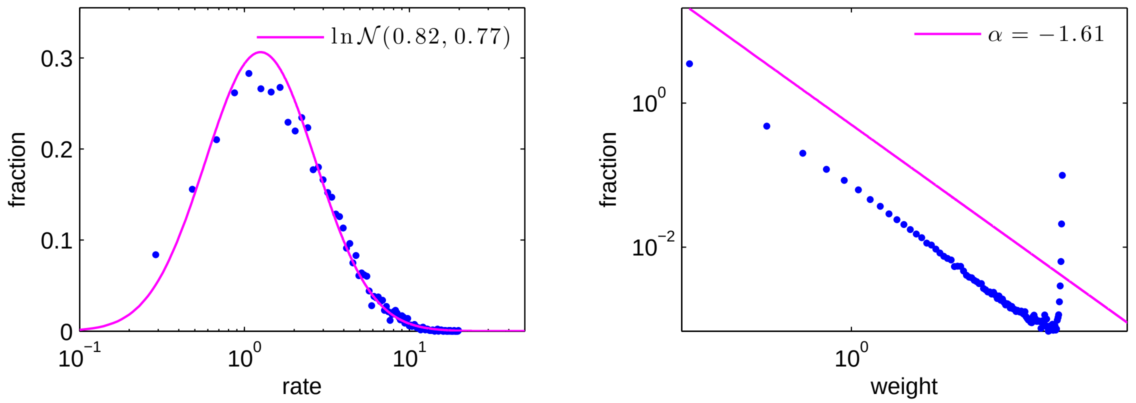

Figure M: Distribution of weights and rates in the topographic network on the torus with additive STDP. Left: Distribution of rates of excitatory neurons, the magenta line represents a log-normal fit. Right: Distribution of the weights of E-E connections, the magenta line represents a truncated power-law fit.

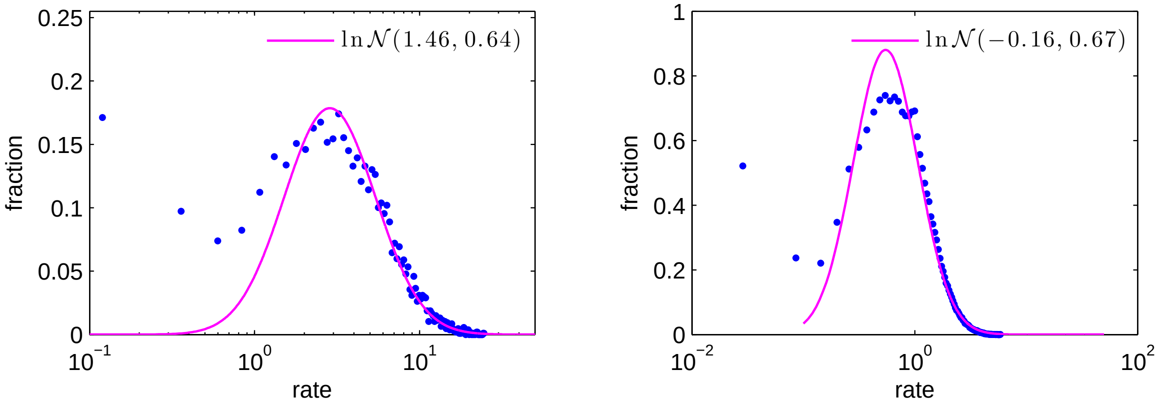

Figure N: Distribution of weights and rates in network with partly multiplicative STDP. Left: Distribution of rates of excitatory neurons. Right: Distribution of the weights of E-E connections. On both panels, the magenta line represents a log-normal fit.

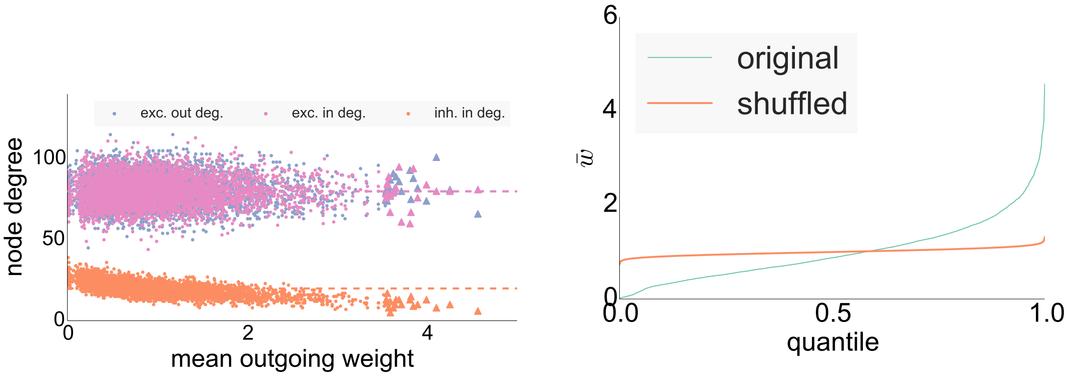

Figure O: Clustering of the synaptic weights in the model with van Rossum STDP. Left: Mean values of outgoing excitatory weights per excitatory cell versus node degrees. Driver neurons shown as triangles. Dashed lines indicate means. Right: Quantiles of distributions of mean outgoing weights of equilibrium network (orange line) and of networks with shuffled synaptic weights (cyan line) averaged over 100 shufflings.

strongest outgoing synapses on cells with higher firing rates (see Fig. R, right), leading to the emergence of driver cells with a high mean outgoing weight.

As in the case for van Rossum STDP, the dynamical effect of driver neurons on their postsynaptic networks is much less pronounced compared to the case of additive STDP, but it is still present.

For the simulations we set $\eta_+ = \eta_- = 10^{-3}$.

12.3 Synaptic scaling

A classical form of homeostatic plasticity called synaptic scaling acts on timescales of hours to days [6]. In the base model, homeostatic plasticity is implemented as synaptic weight normalization at the postsynaptic site of E-E connections. Instead of this homeostatic rule acting at fast time scales we also considered a synaptic scaling acting of the form

$$\frac{dw}{dt} = -\eta(\nu - \nu_0)w^2 \tag{5}$$

acting on much slower time scales that was analyzed in [7], where w denotes the weight of a synapse, ν denotes the firing rate of the postsynaptic cell (taken as a running average over some time window having a length of a few seconds or minutes), ν_0 is a target firing rate and η a learning rate.

For the simulations we performed the updates according to Eq. 5 at fixed time intervals of 50 ms and chose a learning rate $\gamma = 10^{-6}$ along with $\nu_0 = 0$ Hz. Firing rates were computed using a sliding window of length 100 s. Note that the target rate is never attained by any of the cells due to external and recurrent input. This setup yields similar weight distributions and clustering characteristics of strong synapses that are comparable to the base model (see Fig. T).

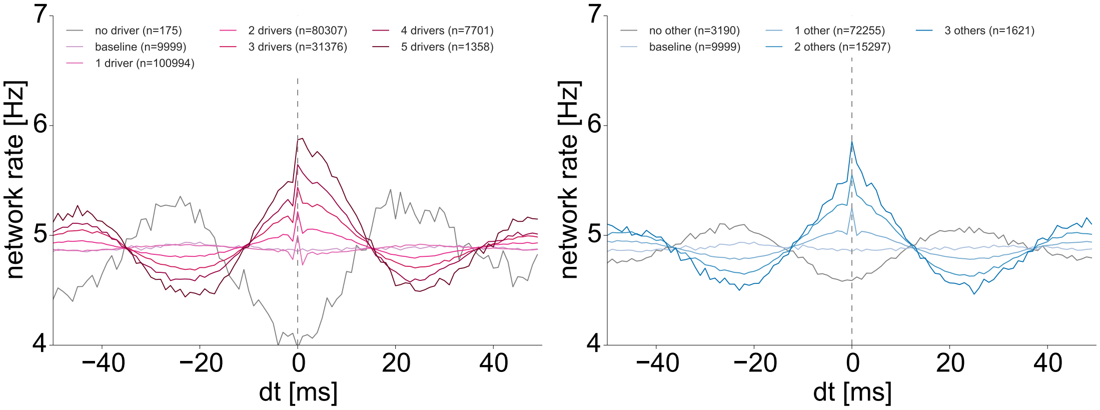

Figure P: Spike triggered averages for joint driver spiking (left) and joint random non-driver spiking (right) in a network with van Rossum STDP. Solid lines show excitatory population firing rate, dashed line indicates 2 ms time bin containing joint spike event.

Configuration	μ	σ	% strong synapses	% strong synapses from drivers
additive	1.0	2.74	2.37	52.49
additive, slow homeostasis	1.0	1.25	1.43	52.43
additive, no iSTDP	1.0	2.73	2.13	23.10
van Rossum	1.0	0.68	1.46	81.26
multiplicative	1.0	0.62	1.12	82.47

Table 1: Statistics of strong synapses for different network configurations.

We observe that in this altered model convergence of synaptic weights takes much longer than in the base model (convergence took circa $15,000$ s in the example).

13 Clustering of strong synapses

In order to more closely examine the clustering of strong outgoing synapses at single cells, we define a class of *strong synapses* as the excitatory synapses having efficacies larger than $\mu + 3\sigma$ where μ and σ denote the mean and standard deviation of all excitatory synapses, see Tab. 1. We subsequently calculate the fraction of strong synapses at each cell and show histograms of these distributions for various configurations in Fig. U. Note that the tail in the network without inhibitory STDP is much smaller than in the case of a network including inhibitory STDP. The clustering effect is even more pronounced for the cases of van Rossum and multiplicative STDP rules.

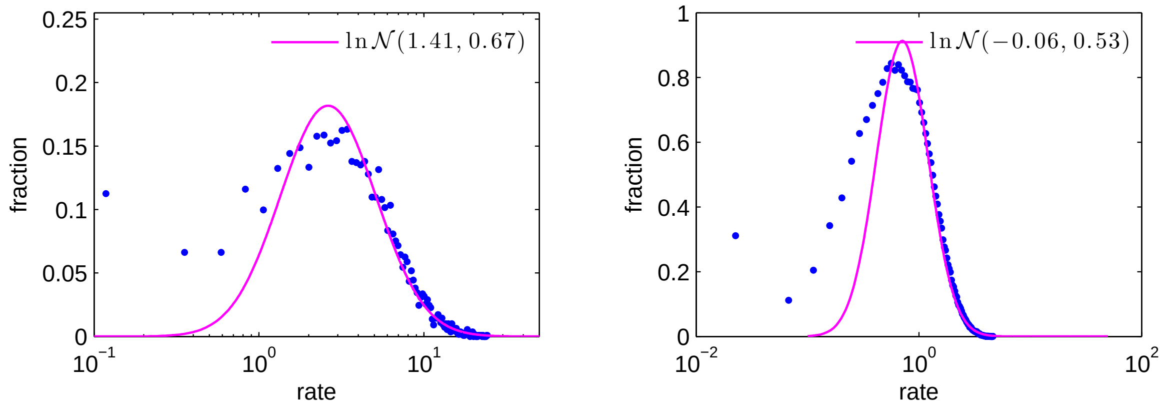

Figure Q: Distribution of weights and rates in network with multiplicative STDP. Left: Distribution of rates of excitatory neurons. Right: Distribution of the weights of E-E connections. On both panels, the magenta line represents a log-normal fit.

14 Derivation of stationary inhibitory weights

We derive here the stationary weight of an inhibitory STDP synapse as described in Eq. 2 of the main text. The stationary weight can be obtained by solving $\langle \Delta w \rangle = 0$,

$$
\begin{aligned}
&\nu_0 \tau \left[A_+ \left(e^{-\frac{w}{\nu_0 \tau (V_{\text{thres}} - V_{\text{rest}})}} - e^{-\frac{w + V_{\text{thres}} - V_{\text{rest}}}{\nu_0 \tau (V_{\text{thres}} - V_{\text{rest}})}} \right) + A_- \left(e^{-\frac{1}{\nu_0 \tau}} - 1 \right) \right] = 0 \\
\Leftrightarrow\ & A_+ e^{-\frac{w}{\nu_0 \tau (V_{\text{thres}} - V_{\text{rest}})}} \left(1 - e^{-\frac{1}{\nu_0 \tau}} \right) + A_- \left(e^{-\frac{1}{\nu_0 \tau}} - 1 \right) = 0 \\
\Leftrightarrow\ & A_+ e^{-\frac{w}{\nu_0 \tau (V_{\text{thres}} - V_{\text{rest}})}} - A_- = 0 \\
\Leftrightarrow\ & \frac{A_+}{A_-} = e^{\frac{w}{\nu_0 \tau (V_{\text{thres}} - V_{\text{rest}})}} \\
\Leftrightarrow\ & w = \log \left(\frac{A_+}{A_-} \right) \nu_0 \tau (V_{\text{thres}} - V_{\text{rest}}),
\end{aligned}
$$

what yields Eq. 2 of the main text.

References

[1] Vogels TP, Abbott LF (2005) Signal propagation and logic gating in networks of integrate-and-fire neurons. The Journal of Neuroscience 25: 10786–95.

[2] van Rossum MC, Bi GQ, Turrigiano GG (2000) Stable Hebbian learning from spike timing-dependent plasticity. The Journal of Neuroscience 20: 8812–21.

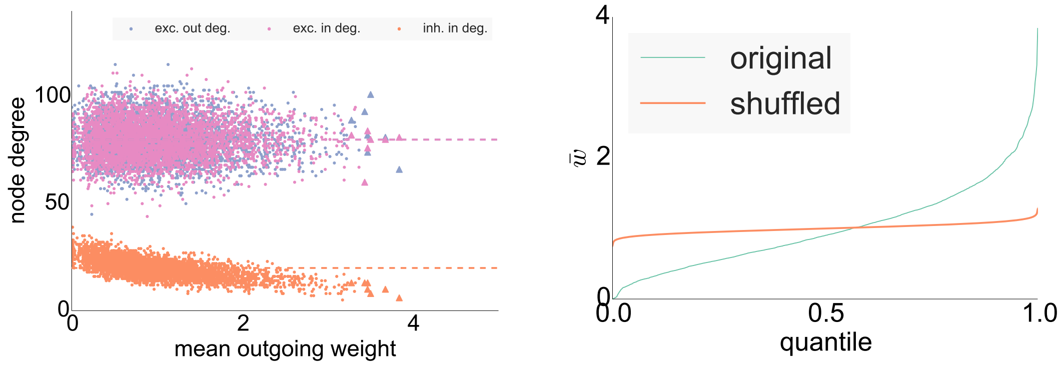

Figure R: Clustering of the synaptic weights in the model with multiplicative STDP. Left: Mean values of outgoing excitatory weights per excitatory cell versus node degrees. Driver neurons shown as triangles. Dashed lines indicate means. Right: Quantiles of distributions of mean outgoing weights of equilibrium network (orange line) and of networks with shuffled synaptic weights (cyan line) averaged over 100 shufflings.

[3] Brunel N, Sergi S (1998) Firing frequency of leaky intergrate-and-fire neurons with synaptic current dynamics. Journal of Theoretical Biology 195: 87–95.

[4] Renart A, Brunel N, Wang XJ (2003) Mean-Field Theory of Irregularly Spiking Neuronal Populations and Working Memory in Recurrent Cortical Networks. In: Feng J, editor, Computational Neuroscience: A Comprehensive Approach, CRC Press, Boca Raton. pp. 431–490.

[5] Voges N, Schüz A, Aertsen A, Rotter S (2010) A modeler's view on the spatial structure of intrinsic horizontal connectivity in the neocortex. Progress in Neurobiology 92: 277–292.

[6] Turrigiano GG, Leslie KR, Desai NS, Rutherford LC, Nelson SB (1998) Activity-dependent scaling of quantal amplitude in neocortical neurons. Nature 391: 892–6.

[7] Tetzlaff C, Kolodziejski C, Timme M, Wörgötter F (2011) Synaptic scaling in combination with many generic plasticity mechanisms stabilizes circuit connectivity. Frontiers in Computational Neuroscience 5: 47.

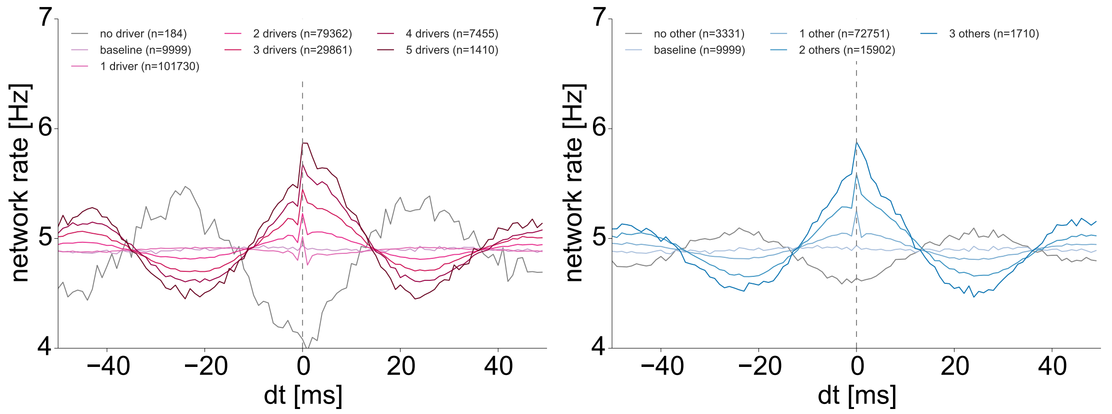

Figure S: Synchrony triggered averages for driver neurons (left) and group of 20 randomly selected non-driver neurons (right) in a network with fully multiplicative STDP. Traces are averages of the excitatory population firing rate and a vertical dashed line marks the 2 ms time bin of synchronous spiking.

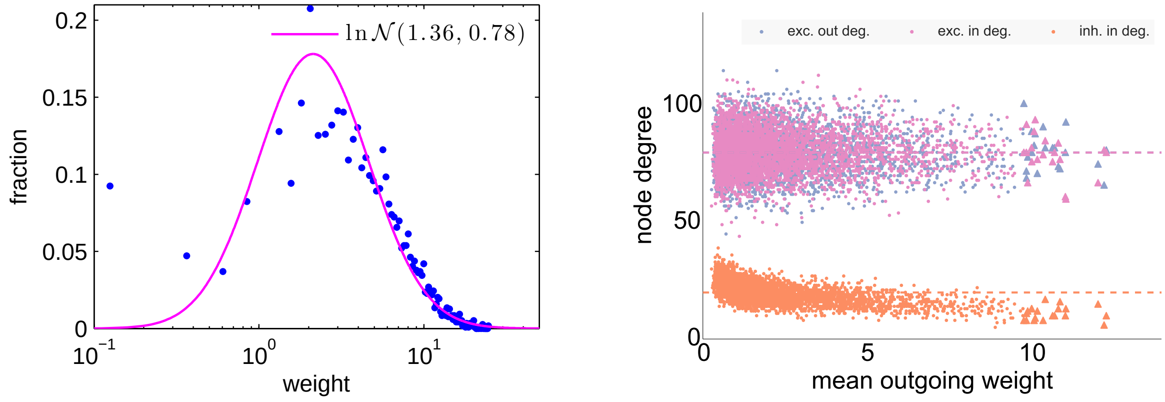

Figure T: Network with synaptic scaling. Left: Firing rate distribution. Right: Mean values of outgoing excitatory weights per excitatory cell versus node degrees in model with synaptic scaling. Driver neurons shown as triangles. Dashed lines indicate means.

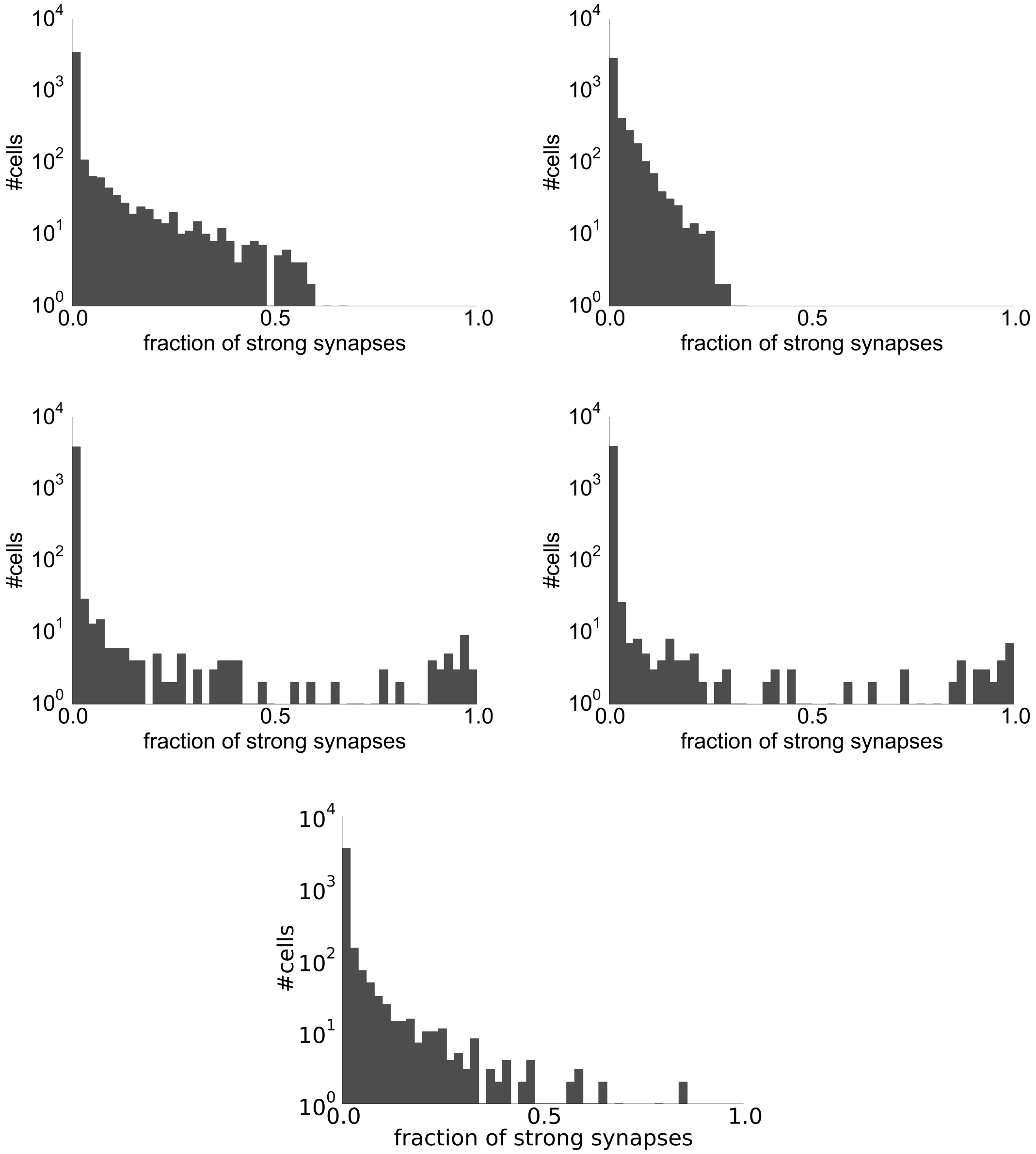

Figure U: Distribution of fraction of strong synapses diverging from excitatory cells for different networks. Network with additive excitatory STDP and additive inhibitory STDP referred to as base network. Top left to bottom right: base network, base network without inhibitory STDP, base network with van Rossum STDP, base network with multiplicative STDP, base network with slow homeostasis.
